# Direct Quantitative Immunochemical Analysis of Autoinducer Peptide IV for Diagnosing and Stratifying *Staphylococcus aureus* Infections

**DOI:** 10.1021/acsinfecdis.1c00670

**Published:** 2022-02-17

**Authors:** Enrique-J. Montagut, Gerardo Acosta, Fernando Albericio, Miriam Royo, Gerard Godoy-Tena, Alicia Lacoma, Cristina Prat, Juan-Pablo Salvador, María-Pilar Marco

**Affiliations:** † Nanobiotechnology for Diagnostics (Nb4D), Department of Surfactants and Nanobiotechnology, Institute for Advanced Chemistry of Catalonia (IQAC) of the Spanish Council for Scientific Research (CSIC), 08750 Barcelona, Spain; ‡ CIBER de Bioingeniería, Biomateriales y Nanomedicina (CIBER-BBN), 28029 Madrid Spain; § Multivalent Systems for Nanomedicine (MS4N), Department of Surfactants and Nanobiotechnology, Institute for Advanced Chemistry of Catalonia (IQAC) of the Spanish Council for Scientific Research (CSIC), 08750 Barcelona, Spain; ∥ Servei de Microbiologia, Hospital Universitari Germans Trias i Pujol, Institut d’Investigació Germans Trias i Pujol, Universitat Autònoma de Barcelona, 08916 Badalona, Spain; ⊥ CIBER de Enfermedades Respiratorias (CIBERES), 28029 Madrid, Spain; # Julius Center for Health Sciences and Primary Care, University Medical Center Utrecht, Utrecht University, 3584 Utrecht, the Netherlands; ¶ Department of Organic Chemistry, Faculty of Chemistry, University of Barcelona, 08028 Barcelona, Spain; ∇ School of Chemistry and Physics, University of KwaZulu-Natal, 4000 Durban, South Africa

**Keywords:** quorum sensing, autoinducer peptides, Staphylococcus aureus, agr group, antibodies, diagnostic

## Abstract

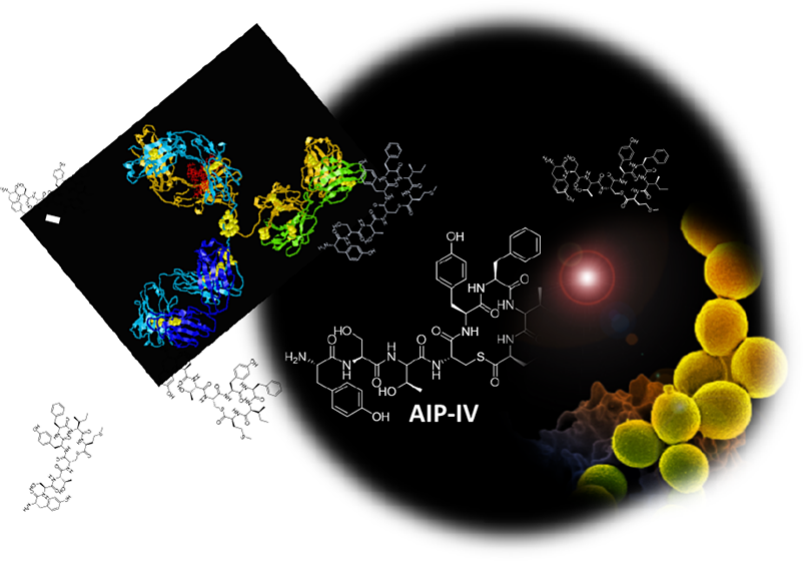

An immunochemical strategy to detect and quantify AIP-IV, the quorum sensing (QS) signaling molecule produced by *Staphylococcus aureusagr* type IV, is reported here for the first time. Theoretical calculations and molecular modeling studies have assisted on the design and synthesis of a suitable peptide hapten (AIPIVS), allowing to obtain high avidity and specific antibodies toward this peptide despite its low molecular weight. The ELISA developed achieves an IC_50_ value of 2.80 ± 0.17 and an LOD of 0.19 ± 0.06 nM in complex media such as 1/2 Tryptic Soy Broth. Recognition of other *S. aureus* AIPs (I–III) is negligible (cross-reactivity below 0.001%), regardless of the structural similarities. A pilot study with a set of clinical isolates from patients with airways infection or colonization demonstrates the potential of this ELISA to perform biomedical investigations related to the role of QS in pathogenesis and the association between dysfunctional *agr* or the *agr* type with unfavorable clinical outcomes. The AIP-IV levels could be quantified in the low nanomolar range in less than 1 h after inoculating *agr* IV-genotyped isolates in the culture broth, while those genotyped as I–III did not show any immunoreactivity after a 48 h growth, pointing to the possibility to use this technology for phenotyping *S. aureus*. The research strategy here reported can be extended to the rest of the AIP types of *S. aureus*, allowing the development of powerful multiplexed chips or point-of-care (PoC) diagnostic devices to unequivocally identify its presence and its *agr* type on samples from infected patients.

*Staphylococcus aureus* is a threatening pathogen and is the leading cause of a broad spectrum of infective processes such as endocarditis, pneumonia, skin and soft tissue infections, osteoarticular infections, and device-related infections. Up to 60% of human population^1^ is colonized by this Gram-positive bacterium^2^ that shows a particular ability to evade the primary innate immune response^3,4^ and therefore acts as a very efficient infective agent. It belongs to the so-called ESKAPE group of pathogens (*Enterococcus faecium*, *S. aureus*, *Klebsiella pneumoniae*, *Acinetobacter baumannii*, *Pseudomonas aeruginosa*, and *Enterobacter* spp.) which include six microorganisms causing nosocomial infections and exhibit extreme virulence and multidrug resistance behavior.^5^ Particularly, methicillin-resistant *S. aureus* (MRSA) is estimated to account for 25% of the *S. aureus* strains with a prevalence of up to 50% in some areas, generating a social and economic burden by means of both community-acquired infections (CAIs) and healthcare-associated infections (HAIs).^6−8^*S. aureus* is one of the earliest pathogens isolated from the airways of cystic fibrosis patients, being positive for more than 70% of neonates and 45% of those becoming persistently colonized.^9^*S. aureus* is also frequently involved in ventilator-associated pneumonia, complicating these infections due to its virulence and antibiotic resistance, leading to high morbidity and mortality rates.^10−12^

The current techniques used to assess the etiology of lower respiratory tract infections often provide poor or inaccurate results aggravated, in the case of *S. aureus*, by the existence of small colony variants (SCVs), which are frequently overlooked or misidentified in culture plates.^13,14^ In addition, culture diagnostic techniques may take up to 72 h to identify the microorganism causing an unaffordable delay to deliver results to the patient, which prompts the use of broad-spectrum antibiotics contributing to the generation of antimicrobial resistance (AMR).^15^ There is a clear and urgent need of finding novel diagnostic approaches for fast and accurate pathogen identification to complement the current diagnostic methods for infectious diseases. Quantitative real-time PCR, focused on the identification of particular genome sequences, and matrix-assisted laser desorption ionization-time-of-flight-mass spectrometry (MALDI-TOF-MS), based on the analysis of the mass distribution of bacterial proteins, are able to more rapidly unequivocally identify *S. aureus*(16−18) and other pathogens. However, these approaches often still require prior culture enrichment steps, expensive equipment, highly trained personnel, and extensive validation for clinical interpretation of the results on routine clinical analyses, which make their wide implementation difficult in all clinical settings or near the patient primary attention centers.^16−18^ An additional unmet challenge of the diagnosis of infectious diseases is the difficulty to differentiate simple carriers from infected patients.^19^ Diagnostic approaches that fulfill the ASSURED (affordability, sensitivity, specificity, user-friendliness, rapidity and robustness, no equipment needed, and deliverable to end users)^20^ criteria are a recognized unmet need for many diseases and particularly to diagnose infections. Implementation of point-of-care (PoC) devices and diagnostic methods accomplishing such criteria would substantially improve and facilitate the management of infectious diseases. A key aspect in achieving this goal is to select appropriate specific biomarker targets whose detection clearly points to the pathogen causing the disease.

The quorum sensing (QS), a population-dependent bacteria communication system that controls the genetic expression of virulence factors and crucial survival mechanisms during pathogenesis, has attracted the attention of the scientific community.^21^ This communication process relies on the release of low molecular weight signaling molecules to the extracellular matrix, whose concentration will increase as a function of population density.^22^ QS in Gram-negative bacteria depends on characteristic molecules such as homoserine lactones (HSLs) called autoinducers (AI), while in Gram-positive bacteria, these AIs are called peptides (AIP, autoinducer peptide).^23^*S. aureus* virulence is under the control of a primary QS system called Agr (accessory gene regulator).^24^ It consists of two transcriptional units, RNAII (containing the genes *agrA*, *agrB*, *agrC*, and *agrD*, transcribed according to the following order *agrBDCA*), responsible of its own autoinduction, and RNAIII, which encodes a series of processes related to virulence. Indeed, the *agr* operon regulates over 70 genes, 23 of which control its pathogenicity and invasive infections.^25^ The activation of the *agr* loci switches the bacterium from being a sessile colonizer to a hostile, invasive pathogen.^26^ Moreover, *S. aureus* can be stratified into four different groups (agr I, agr II, agr III, and agr IV) according to the sequences of the *agrC* and *agrD* genes, which results in four different AIs, which are small (7–9 amino acids) cyclic thiodepsipeptides (the C-terminal carboxylic group forms a thiolactone with the thiol of a Cys) produced in a strain-specific manner.^27^ There have been reported clear associations between the *agr* type and virulence, the ability to form biofilms, and AMR profile.^28−34^ Hence, higher prevalence of generalized exfoliative syndromes or osteoarticular infections has been associated with *agr* group IV,^35,36^ TSS toxin1-producing isolates have been found to belong mainly to *agr* group III, and higher risk of endocarditis has been associated with infections from *agr* types I and II.^37,38^ Other interesting associations have also been recently reported on a study performed with 833 *S. aureus* strains (785 bacteremia and 48 colonizing strains) collected in Spain over a period of 15 years (2002–2017), pointing at the higher prevalence of *agr* IV on colonizing strains, *agr* II on HAIs, and *agr* I on CAIs, while *agr* II would be more prevalent in adults, and *agr* III would be associated with infections in children.^39^ On the other hand, it has also been reported that a large proportion of clinical isolates are consistently found to have a mutationally inactivated Agr system (Agr-negative), which have a survival advantage in the host. However, it has recently been reported that a minor fraction of these Agr-negative mutants can revert their Agr activity upon phagocytosis.^26^ Therefore, *agr* typing could be of great interest to manage infections and distinguish between colonization and infection.^40−44^

Some authors have pointed at the possibility to use AIs as specific biomarkers of infection,^45^ but although AIP-I has been quantified in bacterial cultures using mass spectrometry with limits of detection (LOD) in the low micromolar range,^46,47^ to our knowledge, direct quantification in clinical samples has not yet been achieved. The possible reasons for this fact could be their potential low concentration levels in body fluids or the lack of chemical stability of these AIPs in certain media. Hence, the instability of thiolactone functionality and the possibility of protease degradation of these peptides during the clean-up, extraction, or preconcentration procedures have been reported to be between of the reasons because chromatography and certain bioanalytical techniques have failed to directly detect AIPs in clinical samples.^45^ In this context, the potential of immunochemical techniques to detect and quantify a whole variety of molecules at low concentration levels is well known, even in complex media without the need of previous extraction or clean-up steps, because of the extraordinary properties of the antibodies, which are able to bind antigens with high affinity and specificity, despite the presence of other substances. The final aim of this research line is to go deeper into the role of AIPs and *agr* types in pathogenesis providing antibodies and immunochemical tools for their investigation. Moreover, immunochemical analytical tools would allow quantifying AIPs in clinical samples and validating their value as biomarkers of *S. aureus* infection. As a starting point, we have focused on AIP-IV based on the association of *agr* IV to colonizing strains found by Pérez-Montarelo et al. on the study mentioned above,^39^ which if confirmed, could allow discriminating infection from colonization, preventing unnecessary antibiotic treatment.^39^

## Results and Discussion

### AIP-IV Immunizing Hapten Design

The production of antibodies against low molecular weight targets has several decisive steps, among which, the rational design of the immunizing hapten and its chemical synthesis should be highlighted. Knowledge of the chemical features of the target is a key issue, and in this case, a main challenge was the reported lack of stability associated with the thiolactone chemical function closing the peptide ring (see the AIP-IV structure in Figure 2).^48^ Hence, Park and co-workers^49^ attempted to produce antibodies against AIP-IV by synthesizing a hapten with a lactone group instead of a thiolactone due to its better stability. The antibodies produced against the lactone hapten were used for therapeutic purposes but were never assessed to detect and quantify AIPs in complex biological media or for diagnostic purposes. However, it should be noticed that the lactone functionality used is not exempt from the risk of hydrolysis. In fact, persecuting a similar goal, Debler and co-workers^50^ used a lactam hapten to produce antibodies against HSLs, which are QS signaling molecules produced by Gram-negative bacteria. In light of these precedents, the possibility of using a more stable AIP-IV lactam hapten instead of a lactone to raise antibodies was considered. However, before, we wanted to evaluate the impact that substituting the sulfur atom by a nitrogen atom could have in the electronic and structural properties of the molecule.

**Figure 1:**
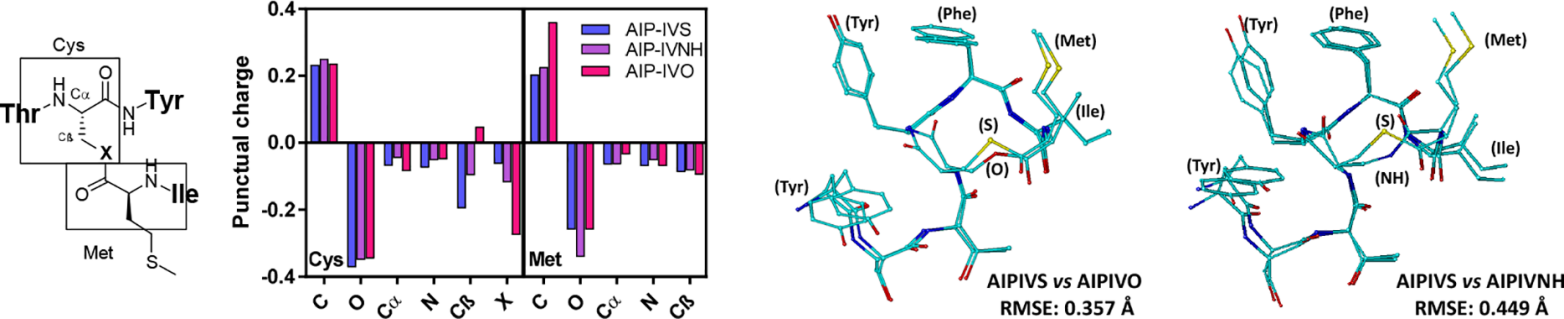
Left: Graph showing the variations in the punctual charges of the amino acid residues affected by the thiolactone, lactam, or lactone functionalities of the proposed immunizing haptens. Right: Structural alignment for AIP-IVS with AIP-IVO and AIP-IVNH molecules where the RMSE values were obtained.

Theoretical calculations and molecular modeling studies are powerful instruments for designing and evaluating the suitability of the hapten chemical structures to raise high-affinity antibodies. Using these tools, it is possible to ensure that the electronic configuration and the geometrical conformation of the hapten mimic as much as possible that of the analyte^51−54^ and also to assess conformational changes on hapten–carrier bioconjugates.^55^ Hence, the minimum energy conformers of the native AIP-IV structure (thiolactone) and of the corresponding lactam or lactone AIP-IV structures were calculated using Molecular Mechanics MM+, followed by a semiempirical PM3 method. Regarding the electronic distribution, aside from the “X” functionality, significant differences were only encountered in the region close to the thiolactone/lactam functionalities (see the graph in Figure 1). As it can be observed, the position Cβ, corresponding to the Cys residue of the native AIP-IV that is directly coupled to S, NH, or O in the corresponding AIPIV, AIPIV(NH), and AIPIV(O) molecules, is the only one affected probably due to the fact that while NH and O have donor electron-transfer features, the sulfur atom has acceptor electron properties. The variation is particularly evident in the Cβ position of AIPIV(O), changing the electronegativity of its punctual charge. In respect to the geometry, the impact of changing the thiolactone functionality by a lactam or by a lactone was estimated calculating the RMSE (root-mean-square error) by overlapping the lactam or the lactone with the native thiolactone peptide (see models in Figure 1). The RMSE values of 0.449 and 0.357 Å were recorded for the lactam and the lactone molecules, respectively. It should be noticed that different conformations of the native AIP-IV at their minimum energy level showed RMSE values of just 0.094 ± 0.062 Å. On the other hand, certain chemical properties, such as its capability to establish hydrogen bonds with other molecules, pointed at major differences for the lactam molecule, in respect to thiolactone or lactone. Despite these results, the risk of instability of lactone and the precedents of Debler and co-workers^50^ with the HSLs prompted us to select the lactam as the stable alternative for the thiolactone functionality of the AIP-IV immunizing hapten (AIPIVNH hapten). Even though, owing to the conformational and chemical differences of the lactam presented above, the synthesis of a hapten preserving the thiolactone moiety (AIPIVS hapten) was also addressed.

**Figure 2:**
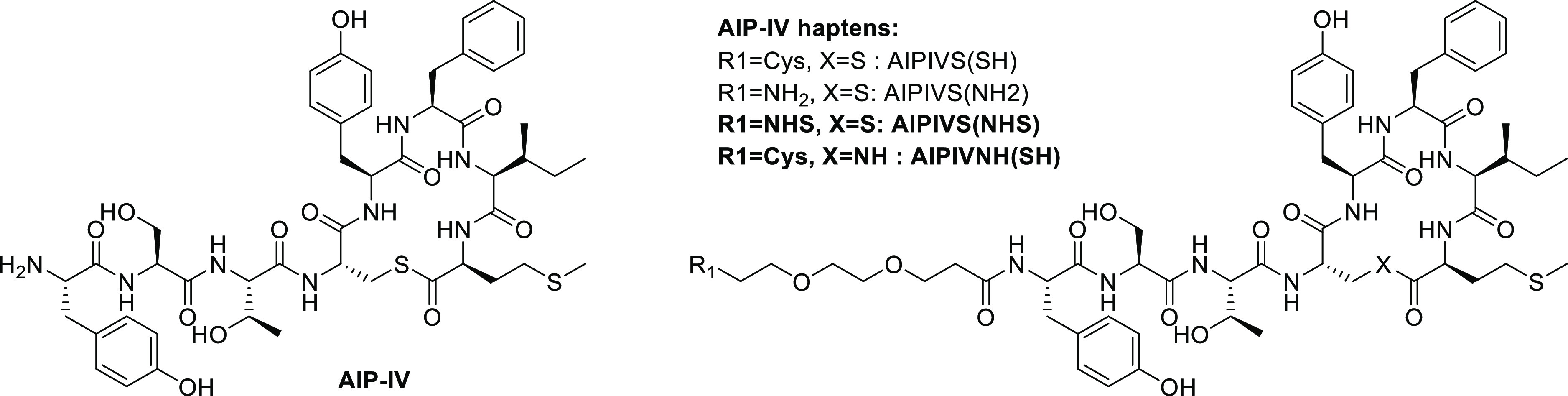
Chemical structures of the native AIP-IV and the AIP-IV haptens designed. In bold, those that were finally used to raise antibodies against the *S. aureus* QS signaling molecule AIP-IV.

The immunizing haptens designed incorporated a short bifunctional oligo ethylene glycol (8-amino-3,6-dioxaoctanoic acid, O2Oc) spacer arm at the N-terminal Tyr residue of the AIP-IV (see Figure 2). The amino end of O2Oc could be adequately modified for further protein bioconjugation. The hapten maximized the exposure of the cyclic peptide structure to the immune system, which is the most characteristic molecule moiety. Moreover, the ethylene glycol units of the spacer arm ensured hydrophilicity while minimizing its antigenicity.

### General Strategy for the Synthesis of Native AIPs (I–IV) and the AIP-IV Haptens

The synthesis of the AIP-IV hapten and the native AIP (I–IV) target analytes started with the common linear peptide precursor (see Figure S1), synthesized using a fluorenymethoxycarbonyl (Fmoc)/*tert*-butyl (*t*Bu) solid-phase peptide synthesis strategy on a 2-chloro-trityl chloride (CTC) resin. All functional groups of the amino acid side chains were protected with *t*Bu with the exception of the thiol group of the internal Cys residue that will form thiolactone, which was protected with a 4-methoxyltrityl group (Mmt). For the case of AIP (I–IV) target analytes, a *tert*-butyloxycarbonyl (Boc) protecting group was used for the α-amino group of the N-terminal amino acid (Tyr) of each linear peptide precursor. The AIP (I–IV) linear peptidyl resins were treated with a low concentration of trifluoroacetic acid (TFA) [TFA/H_2_O/DCM (2:4:96)] to remove the Mmt from the Cys and to cleave the peptide from the CTC resin, maintaining intact Boc/*t*Bu protecting groups. Then, the linear precursor peptides were treated with benzotriazol-1-yloxytripyrrolidinophosphonium hexafluorophosphate (PyBOP)/*N*,*N*-diisopropylethylamine (DIEA) to form the thioester between the thiol of the internal Cys and the carboxylic acid of the C-terminal. Finally, Boc/*t*Bu protecting groups were eliminated by standard acidolysis treatment (TFA/triisopropylsilane (TIS)/H_2_O, 95:2.5:2.5).

### Synthesis of the AIPIVS(X) Haptens

For the first AIP-IV hapten proposed (AIPIVS(SH)), Boc-Cys(Trt)-OH was incorporated at the O2Oc end with the idea to perform an orthogonal bioconjugation with the proteins previously derivatized with maleimide. The protected peptide was cleaved from the solid support with TFA/H_2_O/DCM (2:4:96), without deprotecting the Trt of the Cys N-terminal, and cycled to form thiolactone in solution as mentioned above. Unfortunately, the global deprotection with TFA/TIS/H_2_O (95:2.5:2.5), which removes the Trt of the Cys terminal and the Boc/*t*Bu groups, rendered two compounds, the expected AIPIVS(SH) hapten and a secondary product resulting from the attack of the thiol group of the N-terminal Cys residue to thiolactone, giving rise to a large thioester peptide cycle (see Figure S2, top) according to NMR. To overcome this problem, it was proposed using a less nucleophilic amino group (AIPIVS(NH_2_) hapten), instead of an SH, for bioconjugation to the proteins. However, also in this case, the formation of the undesired more stable large lactam cyclic peptide, resulting from the reaction of the amine group with thiolactone, was observed when the protecting groups were eliminated (see Figure S2, bottom).

In light of these results, it was clear that the functional group for bioconjugation did not have to have a nucleophilic characteristic, for which reason a third hapten with a carboxylic group in the form of NHS (*N*-hydroxysuccinimide) ester was proposed. Thus, the AIPIVS(NHS) hapten was prepared by the reaction of AIP-IV with 3,6-dioxaoctandioic acid bissuccinimidyl ester (dihydroxysuccimidyl-PEG (2) ester) (see Figure S1), without further difficulties, and obtained with 96% purity according to UV and HPLC–MS analyses. The NHS ester could be directly used to form amide groups with the Lys residues of the protein and, as it has an electrophilic character, did not interfere reacting with the labile thiolactone.

### Synthesis of the AIPIVNH(SH) Hapten

The AIP-IV-NH (SH) hapten lactam derivative (see Figure S3) was synthesized using a Fmoc-Dap(Alloc)-OH, instead of a Fmoc-Cys(Mmt)-OH, and the AIPIV linear precursor was elongated with a Boc-Cys-(Trt) at the end. Prior to the cleavage of the linear precursor from the peptidyl resin, the Alloc group was eliminated by treatment with tetrakis(triphenylphosphine)-palladium(0) (Pd(Ph_3_P)_4_) and phenylsilane (PheSiH_3_). Finally, the lactam ring was also formed using PyBOP/DIEA, and the side-chain protecting groups, including the Trt of the Cys, were removed with a high TFA content solution to obtain the desired hapten AIP-IV-NH(SH) with 98.4% purity according to UV and HR-MS.

### Antibody Development

Bioconjugation of the hapten AIPIVS(NHS) to HCH and BSA was carried out by just adding the hapten to the solutions of the proteins in PBS. A direct nucleophilic reaction of the amino groups of the Lys residues with the succinimide ester moiety of the hapten allowed us to obtain easily the corresponding AIPIVS-HCH and BSA bioconjugates. Bioconjugation of the AIPIVNH(SH) hapten was performed through a two-step procedure by first reacting the Lys residues of the proteins with succinimidyl iodoacetate (SIA), to introduce iodine as a living group, and subsequently linking the hapten through a selective reaction of the SH group of the terminal Cys residue. MALDI-TOF-MS analyses of the corresponding AIPIVS(NHS)- and AIPNH(SH)-BSA bioconjugates recorded hapten densities of 8 and 6, respectively (see Table S1). Antisera were raised against AIPIVNH(SH)-HCH (As376, As377, and As378) and AIPIVS(NHS)-HCH (As379, As380, and As381) using the standard immunization protocol described in the experimental section.

### Development of AIP-IV (As380/AIPIVS(NHS)-BSA) ELISA

The avidity of the antisera against homologous and heterologous BSA bioconjugates (in respect to the chemical structures of the haptens) was assessed by bidimensional titration experiments from which the most suitable concentrations for each As/bioconjugate competitor combinations were selected. Although not all of them gave rise to usable competitive assays, in general, homologous combinations performed better than the heterologous ones (Table S2). On the other hand, it is relevant mentioning that the antisera raised using the thiolactone-immunizing hapten (AIPIVS(NHS)-HCH) provided immunochemical assays with substantially higher detectability than the antisera obtained immunizing with the lactam hapten (AIPIVN(SH)-HCH). As can be observed in Table S2, the ELISAs using As379 and As380 (against AIPIVS) show lower IC_50_ values (79 and 6 nM, respectively) compared to the ELISAs using As376–378 (188, 360, and 294 nM of IC_50_, respectively). These results agree with the theoretical data discussed above, in which, additionally to the electronic distribution, significant differences in the geometry were predicted for the lactam in respect to thiolactone (RMSE 0.449 Å). Moreover, as mentioned before, there are clear differences in respect to the capabilities to establish hydrogen bond interactions of these two functionalities.

From all the antisera raised against AIPIVS(NHS)-HCH, As380 was giving the best immunoassay features under homologous and standard immunoassay conditions [assay buffer 10 mM PBST (0.05% Tween), 30 min competitive step]. For this reason, As380/AIPIVS(NHS)-BSA was selected for further investigations with the aim of improving its performance and getting knowledge of the behavior of the assay under different physicochemical conditions. With this purpose, the effects of several factors such as pH, ionic strength, concentration of Tween 20, competition, and preincubation time, as well as the concentration of organic solvents such as DMSO, were investigated.

The results of these studies are shown in Figure S10, where it can be observed that the As380/AIPIVS(NHS)-BSA immunochemical assay is quite robust in respect to variations in the pH and concentration of Tween 20 in the assay buffer. Hence, the ELISA As380/AIPIVS(NHS)-BSA performed quite well on a broad range of pH values, keeping the best features between 4.5 and 8.5, and the concentration of Tween20 did not affect substantially the assay if used below 0.05%, although it should be noticed that higher concentrations had a negative effect on the detectability. At pH 9.5, the assay was still usable, although a slight decrease in detectability was observed (IC_50_ 7.98 nM at pH 7.5 vs 13.2 nM at pH 9.5). This fact could be related to the instability of thiolactone at basic pH. Regarding conductivity, a slight improvement in the detectability was observed with the increase of the ionic strength, but a proportional reduction of the maximum absorbance was also produced. The addition of a small percentage of DMSO (2%) to the competitive step slightly increased the detectability, although the slope was slightly affected (data not shown). The competition time was set at 30 min since although the same detectability could be reached at shorter incubation times, the maximum signal of the assay decreased significantly. A slight improvement in assay detectability was observed if the analyte and the antibody were preincubated overnight or just for 10 min at room temperature (RT) (IC_50_ 3.7 and 3.8 nM, respectively, vs 4.9 nM if no preincubation) without affecting the maximum signal. All these results pointed at the robustness of the assay since any analytical parameter was significantly affected but also at the possibility of a slight improvement, if required. However, further studies were conducted without modifying the assay conditions (see Table S3 for a summary of the assay conditions selected for the As380/AIPIVS(NHS)-BSA ELISA).

### Analytical Characterization of the AIP-IV ELISA

The analytical features of the assay run under these conditions are enclosed in Table 1. The assay showed an IC_50_ value of 2.90 ± 0.19 nM and an LOD of 0.22 ± 0.06 nM, a detectability which was below the concentration values found for AIP-I in culture media.^46,47^ The assay attains higher detectability than other methods developed for the quantification of AIPs based on mass spectrometry.^46,47,56−59^ Even though AIPs have never been directly quantified in clinical samples, the excellent features and detectability of the developed assay make foresee its potential for further studies to gain knowledge about the role of AIPs in the development of pathogenesis and to assess the convenience of using AIPs as biomarkers of disease.

**Table 1:** Analytical Features of the As380/AIPIVS (NHS)-BSA ELISA for the Detection of AIP-IV

analytical parameters	PBST	TSB diluted 1/2
*A*_min_	0.15 ± 0.04	0.09 ± 0.01
*A*_max_	1.46 ± 0.01	1.60 ± 0.05
slope	–0.91 ± 0.03	–0.89 ± 0.03
IC_50_	2.89 ± 0.19	2.80 ± 0.17
dynamic range	0.68 ± 0.14 to 14.26 ± 0.93	0.55 ± 0.11 to 12.84 ± 6.20
LOD	0.22 ± 0.06	0.19 ± 0.06
*R*^2^	0.996 ± 0.003	0.995 ± 0.003

The concentrations of the BSA conjugate and As dilution used were 0.63 μg mL^–1^ and 1/4000, respectively. The parameters for the ELISA in Tryptic Soy Broth (TSB) 1/2 refer to the diluted sample. The concentrations are expressed in nM, and the data shown correspond to the average of the analytical parameters recorded from assays performed in three different days using at least two wells/replicates per concentration of the standard curve.

**Figure 3:**
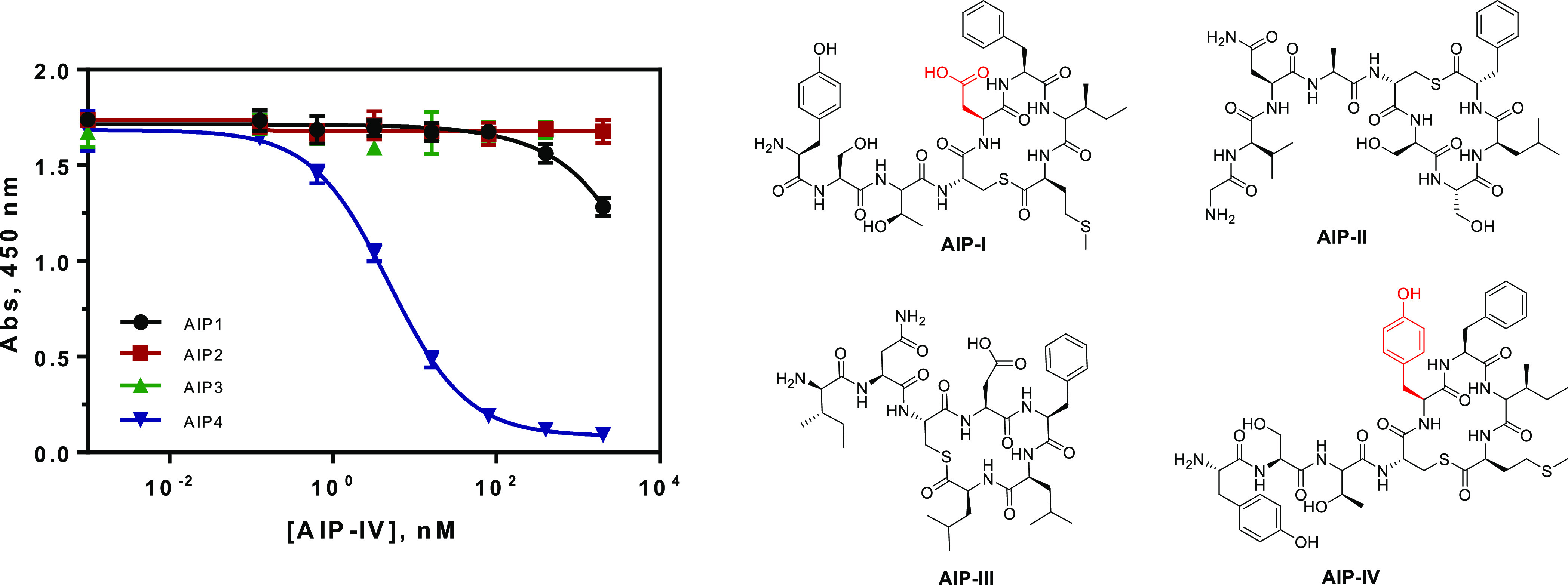
Left: Results from the specificity study in respect to the AIPs of the four *S. aureus* agr groups. AIPs I–IV were used to build standard curves and measure them with the As380/AIPIVS(NHS)-BSA ELISA. The cross-reactivity was less than 0.001% for AIP-I–III. Each calibration point was measured in triplicates on the same ELISA plate, and the results show the average and standard deviation of analysis made on three different days. Right: Chemical structures of the four AIPs. The structural differences between AIP-I and AIP-IV are highlighted in red.

Because of the reported evidence that associate the Agr type with different forms of infection or colonization, assessing the specificity of the As380/AIPIVS(NHS)-BSA ELISA developed in respect to the other *S. aureus* AIPs was of great importance.^60^ Thus, in addition to AIP-IV, AIPs I–III were also synthetized and used to build standard curves that were measured in the ELISA. Surprisingly, despite the great structural similarities (see Figure 3), the ELISA was highly specific for AIP-IV, and the cross-reactivity of other AIPs was less than 0.001% even for AIP-I, which differs from AIP-IV in just one amino acid (see the red moieties of AIP-I and AIP-IV in Figure 3), demonstrating that the tyrosine residue present in the AIP-IV cycle is essential for the biorecognition.

### Implementation of the As380/AIPIVS(NHS)-BSA ELISA to the Analysis of AIP-IV in Culture Media

**Figure 4:**
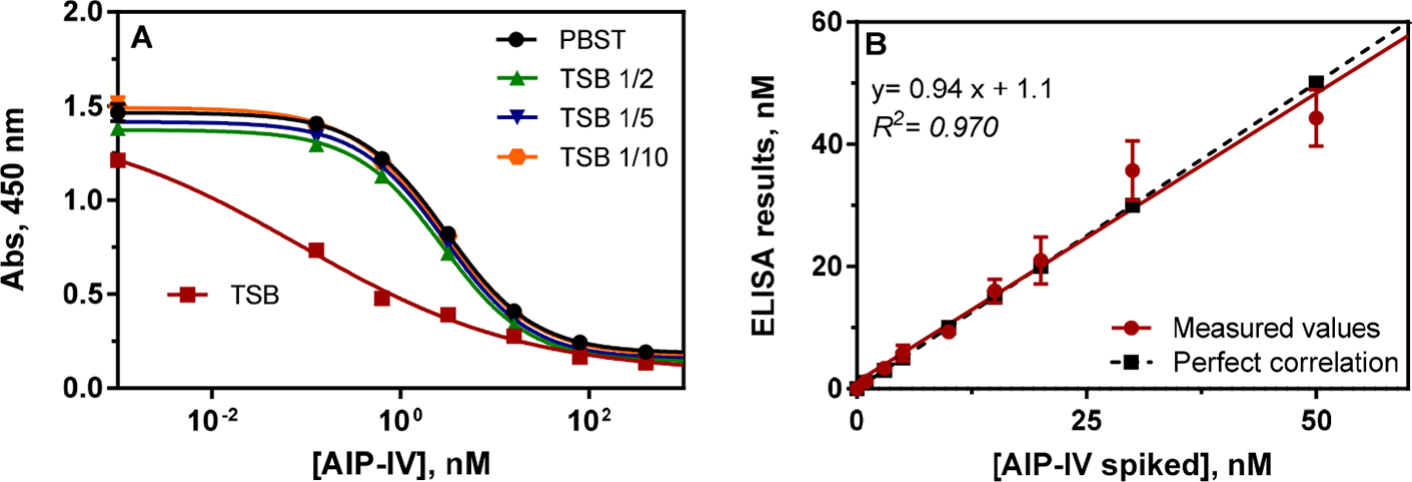
(A) AIP-IV calibration curves using the As380/AIPIVS(NHS)-BSA ELISA competitive indirect assay run in PBST buffer, TSB culture media, and TBS diluted with the buffer at different ratios. The results shown are the average and standard deviations of analysis made on two different days. Each calibration point was measured by duplicates on each day. (B) Graph showing the correlation between the AIP-IV-spiked concentration and the concentration measured with the As380/AIPIVS(NHS)-BSA ELISA. Accuracy experiments were run in TSB culture media diluted 1/2 using PBST. Each calibration point was measured in triplicates on the same ELISA plate, and the results show the average and standard deviation of analysis made on three different days.

The possibility to use this ELISA to directly analyze the profile of release of AIP-IV by *S. aureus* in media culture was investigated. With this purpose, on a first instance, the potential non-specific interferences caused by the TSB were evaluated by building AIP-IV calibration curves in this broth diluted several times with the assay buffer. Figure 4 shows the calibration curves obtained under these conditions. As it is possible to observe, undiluted TSB affects substantially the assay performance; however, a simple one-half dilution with the assay buffer is sufficient to overcome these interferences. Moreover, further dilution of the matrix provided identical curves to the one performed in buffer, which indicates that further dilution does not affect the assay performance.

Table 1 shows the analytical parameters of the calibration curves run in buffer and in 1/2 diluted TSB. The 1/2 dilution provides an immunochemical assay with IC_50_ and LOD values of 5.60 ± 0.34 and 0.38 ± 0.12 nM (originally 2.80 ± 0.17 and 0.19 ± 0.06 nM without taking into account the 1/2 dilution with the assay buffer), which do not compromise the detectability of the assay, considering the reported concentration values of AIPs in the TSB culture broth (i.e., 2–15 μM range^47^). Moreover, the detectability achieved by this ELISA is higher than that reported when using mass spectrometry (MS) methods and does not require any samples pretreatment (see Table S4 for a summary of the detectability of MS reported approaches). Thus, AIP detection by MS has been unwieldy requiring extensive sample clean-up.^56,57^ MALDI-TOF-MS has been used for rapid detection of AIPs from other Gram-positive bacteria, on crude supernatant cultures, but only with qualitative purposes.^58^ Using a hybrid ion trap Orbitrap mass spectrometer (the LTQ Orbitrap), Cech and co-workers were able to quantify AIP in MRSA cultures with a limit of quantification of 2.6 μM,^59^ and Junio and co-workers were able to achieve an LOD of 0.25 μM for AIP-I by directly injecting the culture broth, previously filtered through a 0.22 μm surfactant-free cellulose acetate, on a UHPLC coupled to the LTQ Orbitrap.^47^ More recently, Todd and co-workers were also able to quantify AIP-I using a new generation of a high-resolving power hybrid mass spectrometer using a combination of a segmented quadrupole and an Orbitrap mass analyzer (the Q-Exactive) coupled to an UPLC reaching an LOD and LOQ of 0.0035 and 0.10 μM, respectively,^46^ concentration values that still are higher than those of the ELISA reported in this paper.

The accuracy of the assay was evaluated by measuring a set of TBS blind-spiked samples. As it is shown in Figure 4, a good correlation between the spiked concentrations and the ELISA results was observed with a slope of 0.94, very close to 1, and a regression coefficient of 0.97, pointing at the good accuracy of the assay to quantify AIP-IV in TSB culture broth samples. Moreover, the intra-plate, inter-plate, and inter-day precision of the assay was also very good as it can be observed in Table 2, where the coefficients of variation (CVs) found at three concentrations levels are shown. The results indicated an excellent assay precision, although, as expected, the higher variability was found at the limit of quantitation of the assay (IC_80_), particularly when the assay was performed on different plates or different days (% CV intra-plate, 11.5%; inter-plate, 19%; and inter-day, 34.8%). However, in all other situations, the % CV remained lower, below 10%, demonstrating that the assay can be used to provide reliable quantification data in respect to the AIP-IV concentration levels found in complex matrices.

**Table 2:** Results from the Precision Studies of the ELISA for AIP-IV

	[AIP-IV]	R1	R2	R3	mean	SD	% CV
inter-day	high	12.85	13.79	12.52	13.05	0.66	5.1
	medium	2.64	2.30	2.35	2.43	0.18	7.4
	low	0.43	0.21	0.42	0.35	0.12	34.8
inter-plate	high	14.80	15.12	15.33	15.08	0.27	1.8
	medium	3.12	3.57	3.01	3.23	0.30	9.2
	low	0.83	0.96	0.65	0.81	0.15	19.0
intra-plate	high	14.16	12.27	13.56	13.33	0.96	7.2
	medium	3.33	2.87	2.79	3.00	0.29	9.7
	low	0.77	0.63	0.65	0.68	0.08	11.5

TBS samples for precision studies were prepared by spiking the TBS broth at three levels corresponding to the concentrations providing 80% (low), 50% (medium), and 20% (high) of the maximum signal of the assay in order to cover all the dynamic range. The CV was calculated following the equation CV (%) = σ/μ × 100. The ELISA was run in the TBS culture broth diluted 1/2 with the assay buffer. The TBS samples were measured in triplicate on the same ELISA plate (intra-plate) and on three different days (inter-day) or on three different plates (inter-plate). The concentrations of the replicates, mean, standard deviation, and ICs are expressed in nM; R: replicate; σ: standard deviation; and μ: average.

### Assessment of AIP-IV Production by Clinical Isolates

As a pilot study, a set of clinical isolates from patients infected or colonized with *S. aureus*, according to the current definitions, and showing distinct respiratory *S. aureus* pathologies were selected. The samples were genotyped to obtain knowledge of their *agr* type (see Table 3 for data regarding these samples) and inoculated on TSB. Bacterial growth was similar for all the strains, independent of the *agr* system or type of infection, as evidenced by the measurement absorbance at 600 nm and the OD_600_ at different time intervals for a 25 h period (see Figure S11 and Table S5 for OD_600_ values and cfu mL^–1^ data of each point). Small sample aliquots were taken at different time intervals (1 h, from 0 to 6 h of growth, 3 h, from 6 to 24 h growth, and after 48 h) and measured with ELISA after a 1/2 dilution with the assay buffer, as described above.

**Table 3:** Description of the Clinical Isolates Used in This Pilot Study

# clinical isolate	Agr system	infection type
6_19850	I	pneumonia
3_40448	II	tracheobronchitis
197_63535 (CC45, CC121)^a^	I, I/IV^a^	pneumonia
165_36759	IV	tracheobronchitis
32_75664	III	carrier
48_86474	IV	bronchia colonization
Newman	I	
USA300	I	

a The clinical isolate 197_63535 was found to be formed by two different clonal complexes. While CC45 was genotyped as agr I, CC121 was classified as doubtful I/IV according to the kit used.

**Figure 5:**
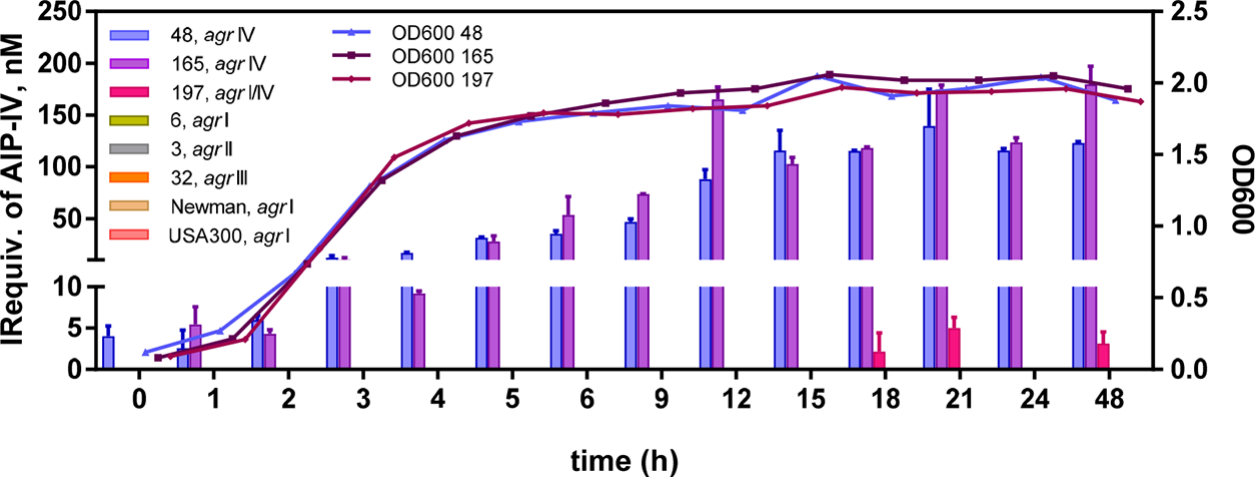
Graph showing the AIP-IV immunoreactivity equivalents (IRequiv) measured at different times from aliquots taken from the culture media where different *S. aureus* strains were grown. Culture broth samples were taken at the selected times, and the AIP-IV IRequiv were measured using the developed As380/AIPIVS(NHS)-BSA ELISA. IRequiv was only detected in the culture broth where clinical isolates genotyped as Agr IV (# 48 and #165) grew. Sample #197 was genotyped as agr I (CC-45); however, the clinical sample was found to contain two different strains, the second one (CC121) was classified as doubtful agr I/IV according to the kit used. Culture media of the rest of the clinical isolates genotyped as Agr I–III (# 6, 3, and 32, Newman and US300) did not show any immunoreactivity. See Table 3 for more information on the features of the samples measured. Each calibration point was measured in triplicates on the same ELISA plate, and the results show the average and standard deviation of analysis made on two different days.

Figure 5 shows the AIP-IV immunoreactivity equivalents (IR equiv) recorded for all the clinical strains. As expected, any AIP-IV IR equiv were detected in the samples from the cultures where strains with *agr* systems I–III were grown. On the other hand, AIP-IV IR equiv of up to 200 nM were recorded for two of the bacterial clinical isolates genotyped as *agr* IV (#48 and #165). Measurable AIP-IV IR equiv could be detected almost immediately after introducing the inoculum in the culture media (strain # 48 at time zero and strain #165 after 1 h) even if the OD_600_ values were below 0.5. Junio et al.^47^ could only detect AIP-I 4 h after inoculating the bacteria in the media culture but the concentration levels recorded were in the micromolar range. The reason for this difference may be diverse, including the *agr* type, the clinical status of the patient from which the bacteria was isolated, or the lower detectability of the methodology employed (LOD 0.25 μM vs 0.38 nM of the ELISA reported here). In contrast, the third clinical isolate (#197), classified as doubtful genotype *agr* I/IV showed a significant lower and irregular production of AIP-IV. Tracing the origin of this sample, it was found that although this patient appeared to be infected by a *S. aureus* strain (197-63538-CC121) showing a regular morphology and behavior, the culture also showed small satellite colonies (197-63538-CC45), which further genotyping studies revealed to be *agr* I. Apparently, the inoculum used was taken from this last type of colonies, which could explain the unexpected behavior of this sample. The fact that after 18 h of growth it was possible to detect some AIP-IV IR equiv still has to be explained and may require further analyses; however, it could have been related to a potential contamination with CC121. Despite the particularities of this last clinical isolate, these results point at the possibility of using this ELISA to identify the *S. aureus* and also to the *agr* strain type in a reliable manner and in a short time.

Further impact of the results reported in this paper could be the use of the technology here presented to identify the dysfunctional *agr* strains by directly quantifying the QS signaling molecules in parallel to real-time PCR^61^ or other indirect conventional methods such as CAMP or VLT (vesicle lysis test) assays, which asses *agr* functionality through the hemolysis effect of δ-lysine.^62^ Hence, several clinical studies point to the higher probability of unfavorable clinical outcomes by invasive infection of dysfunctional agr *S. aureus* strains, pointing to the possibility to use these AIPs as potential biomarkers also to predict prognostic^63,64^ or to distinguish colonization from infection.^62,65,66^ Dysfunctional *agr* has been associated with more abundant biofilm formation, deficient bacteria autolysis, decrease in the antibiotic activity, persistent bacteremia, and higher mortality.^64^ In the same manner, some authors also point to the implication of dysfunctional *agr* system on the formation of *S. aureus* SCV,^67^ often resistant to antibiotics such as gentamicin or aminoglycosides, being able to bypass the effect of other antibiotics.^68^ Thus, *S. aureus* SCVs have been reported to be implicated in chronic infections, with a high capacity for survival and adaptation to the host immunological or inflammatory response.^69^ Although SCVs are not particularly virulent, some phenotypes represent transient states that under certain conditions can revert to wild type or to a different phenotype (distinct from the SCV progenitor), leading to the development of an acute infection.^70,71^ It is well known that during infectious processes, bacteria can switch from planktonic (acute infection) to sessile lifestyle (chronic infection). During this process, the production of toxins, enzymes, and other survival mechanisms is adapted to the needs of the bacterial population, and all these processes may be regulated by the Agr system. Therefore, the technology here reported when extended to other AIPs could be extremely useful to perhaps identify SCVs and to understand all these processes.

Further studies need to be performed in order to implement this technology to the direct analysis of clinical samples and to prove the value of the AIP levels in terms of the severity or the stage of the disease. The data presented in this paper are only preliminary studies addressed to demonstrate the potential of the ELISA developed for diagnostic purposes, whose value may go beyond just identifying the bacteria causing the disease. It will be necessary to carry out a complete well-designed clinical study that takes into consideration all the clinical parameters of interest. However, we can ensure that the As380/AIPIVS(NHS)-BSA ELISA is a highly sensitive, robust, and reliable bioanalytical tool to specifically quantify AIP-IV. Complex biological samples such as culture broth can be directly analyzed without any previous sample treatment, other than diluting the sample two times with the assay buffer. The assay has high sample throughput capability, which has allowed obtaining information about the profile of release of eight different bacterial strains grown in TBS media. The levels of AIP-IV released are in the high nanomolar range and can be measured in less than 1 h of incubation in the culture broth.

The clinical significance of QS molecules needs to be further explored due to the vast set of mechanisms that is under their control. Interesting studies are already addressing the clinical significance and the role of QS molecules from Gram-negative bacteria such as *P. aeruginosa*; for their QS signaling molecules, we have also reported the development of specific antibodies.^72−74^ Similar studies could be addressed in the near future, which may provide interesting information in respect to the involvement of these QS molecules on pathogenesis, as well as the possibility to develop powerful technologies for the diagnostic and surveillance of *S. aureus* infections.

## Conclusions

A highly sensitive and specific immunochemical assay for direct quantification of AIP-IV, the QS signaling molecule specifically released by *agr* IV-type *S. aureus*, has been developed. The excellent analytical features have been accomplished thanks to the high-quality antibodies raised against a molecular modeling-assisted hapten design (AIPIVS), which preserves all the electronic, geometric, and chemical features of the native AIP-IV. Preserving the thiolactone functionality has found to be crucial to achieve a detectability in the low nanomolar range (0.22 ± 0.06 and 0.19 ± 0.06 nM in buffer and in 1/2 TBS, respectively). Hence, a similar hapten in which thiolactone had been substituted by a more stable lactam provided assays with LODs 2 orders of magnitude higher. The assay has shown to be very specific for AIP-IV as demonstrated by cross-reactivity studies and by the measurement of the AIPs produced by *S. aureus* of *agr* types I–III. This fact allows envisaging the possibility to use the technology here reported for phenotyping the *agr*, if antibodies with similar features would be available for each of the AIPs released by the rest of the *agr* groups. AIP-IV could be detected in the low nanomolar range in the culture broth almost immediately (<1 h) after inoculating samples from clinical isolates previously genotyped as *agr* IV. In contrast, any immunoreactivity was detected in TBS samples from media were clinical isolates genotyped agr I–III had been growth under the same conditions.

The antibodies against AIP-IV, reported here for the first time, show great potential for basic research, biomedical investigations, or diagnostic purposes. As is known, there exist a variety of immunochemical analytical configurations in which antibodies can be used, including lateral-flow immunoassays for PoC applications, microarray for multiplexed analysis, or a wide variety of electrochemical or optical biosensors suitable for PoC or for benchtop laboratory analyzers. In light of the results reported here, we envisage addressing our investigations toward the development of specific antibodies against the different AIPs produced by the rest of *agr* types of *S. aureus*. A multiplexed chip combining all these immunoreagents could be a powerful diagnostic tool for identification and surveillance of *S. aureus* infection while stratifying the patients in respect of the *agr* type.

In this paper, we report the development of a microplate-based ELISA which shows great potential for high-throughput sample analyses. More than 100 samples can be simultaneously processed in about 1:5 h. Here, we describe the application of this ELISA to monitor the profile of release of *S. aureus* strains in culture media. Further studies will be addressed to implement the present technology to the direct analysis of clinical samples such as, for example, sputa, BAS, or BAL.

## Methods

### General Methods and Instruments

General methods related to chemical and immunochemical studies performed, as well as the main equipment used, can be found in the Supporting Information.

### Theoretical Calculations and Molecular Modeling

Molecular modeling was performed using the Hyperchem 6.03 software package (Hypercube Inc., Gainesville, FL). Theoretical geometries and electronic distributions were evaluated for AIP-IV hapten derivatives (thiolactone or lactam ring) using semiempirical quantum mechanics MNDO and PM3 models. All calculations were performed using the standard computational chemistry criteria.

### Synthesis of AIPs I–IV and AIP-IV Immunizing Haptens

The experimental procedure for the synthesis of AIPIVS, AIPIVNH, and AIPs I–IV is fully described in the Supporting Information, including the schematic synthetic sequences for each immunizing hapten derivative (see Figures S1–S3) and images of the HPLC-PADs and HR-MS spectra used to characterize the synthesized peptides (see Figures S4–S9).

### Synthesis of the AIP-IV Bioconjugates

#### Lactam Hapten Bioconjugates (AIPIVNH(SH)-BSA and AIPIVNH(SH)-HCH)

A solution of SIA (4.5 μmol) in DMF (0.1 mL) was added dropwise to a solution of the protein (BSA or HCH, 5.6 mg mL^–1^, 0.9 mL in borax/borate buffer), and the mixture was stirred 4 h at RT. The mixture was purified by AKTA using a HiTrap desalting column and borax/borate as eluting buffer to isolate the protein–SIA intermediates. A fraction (20 μL) of the BSA-SIA bioconjugate was kept for MALDI-TOF analysis. Then, a solution of tris(2-carboxyethyl)phosphine (150 μL, 2.5 mg mL^–1^) was added dropwise to the AIPIVNH hapten (3.45 mg, 2.8 μmol) in 1:1 CH_3_CN/H_2_O (150 μL), and the mixture was stirred for 10 min at 40 °C and added (150 μL) to the solutions of proteins (BSA or KLH, 2.8 mg mL^–1^, 1.8 mL in borax/borate buffer). The mixtures were stirred overnight at 4 °C, and on the next day, the bioconjugates were purified by dialysis against 0.5 mM PBS (5 × 5 L) and Milli-Q water (1 × 5 L) and stored freeze-dried at −80 °C. A small fraction (20 μL) of AIPIVNH(SH)BSA was kept for MALDI-TOF analysis, rendering a hapten density of six haptens per molecule of BSA (see Table S1).

#### Thiolactone Hapten Bioconjugates [AIPIVS(NHS)-BSA and AIPIVS(NHS)-HCH]

A solution of the AIPIVS(NHS) hapten (2.9 mg, 2.3 μmol) in anhydrous DMF (0.1 mL) was added dropwise over the protein solutions (BSA or HCH, 2.5 mg mL^–1^, 2 mL in PBS 10 mM buffer), and the mixtures were left to stir for 4 h at RT. Subsequently, the bioconjugates were purified by dialysis against 0.5 mM PBS (5 × 5 L) and Milli-Q water (1 × 5 L) and stored freeze-dried at −80 °C. A small fraction (20 μL) of AIPIVS-BSA was kept for MALDI-TOF analysis, rendering a hapten density of eight haptens per molecule of BSA (see Table S1).

## ELISA

### As380/AIPIVS-BSA ELISA

Microtiter plates were coated with the AIPIVS(NHS)-BSA bioconjugate in coating buffer (0.31 μg mL^–1^, 100 μL/well) overnight at 4 °C and covered with adhesive plate sealers. The next day, the plates were washed with PBST (4 × 300 μL/well), and solutions of the AIP-IV standard (2 μM to 0.13 nM in PBST, 50 μL/well) were added, followed by As380 (dil. 1/4000 in PBST, 50 μL/well), and left without agitation for 30 min at RT. After another washing step, a solution of goat antirabbit IgG-HRP (1/6000 in PBST) was added (100 μL/well) and incubated for 30 min at RT. The plates were washed again, and the substrate solution was added (100 μL) and left for 30 min at RT in the dark. The enzymatic reaction was stopped by adding of 4 N H_2_SO_4_ solution (50 μL/well), and the absorbance was read at 450 nm.

### Immunoassay Evaluation

The performance of the assays was evaluated through the modification of different physicochemical parameters [competence time, incubation time, pH, ionic strength, the presence of a surfactant (% Tween 20), or solubility with the addition of organic solvents] in the competition step.

### Cross-Reactivity Studies

Standard solutions of synthetic AIPs (I–IV) were prepared (0.12 nM to 10 μM in PBST) and measured with the ELISA following the procedure described above. The standard curves obtained were fitted to the four-parameter equation, and the IC_50_ value was used to calculate the cross-reactivity according to the following equation: CR (%) = IC_50_ (cross-reactant)/IC_50_ (analyte) × 100.

### Implementation of the ELISA to the Analysis of Clinical Isolates

#### Description of the Clinical Samples

Six clinical *S. aureus* isolates were retrospectively selected from a collection of strains in the Hospital Universitari Germans Trias i Pujol. Strains were isolated from respiratory specimens obtained from patients under mechanical ventilation admitted at the intensive care unit.^75^ The isolates were stored at −80 °C in a maintenance freezing medium (Oxoid TP, 15731). The strains were identified as *S. aureus* by conventional assays (Gram staining, selective culture media, coagulase test) and antibiotic susceptibility testing and were genotypically characterized by means of a DNA microarray (Clondiag). According to array results, the strains were agr I (culture number 19850), agr II (culture number 40448), agr III (culture number 75664), and agr IV (culture numbers 86474, 63535, and 36759). The clinical isolates and two *S. aureus* reference strains (USA300 and Newman, agr I) were cultured overnight at 37 °C in TSB (5 mL). The next day, dilutions 1/50 in fresh TSB were prepared, and the optical density at 600 nm (OD_600_) was measured. Then, the resulting solutions were shaken at 37 °C until the selected time of growth was completed. When the time was finished, aliquots were extracted for colony forming units (cfus) counting and OD_600_ measurement. Afterward, each solution was centrifuged 10 min at 3000 g, and aliquots were stored at −20 °C for AIP-IV concentration measurement using the ELISA developed in this work. AIP-IV concentrations measured by ELISA are expressed as AIP-IV immunoreactivity equivalents (IRequiv).

#### Matrix Effect Studies

To find out the most appropriate conditions to quantitate AIP-IV in culture samples, AIP-IV standard calibration curves were prepared in the TSB culture broth diluted with PBST (1:2, 1:5, 1:10, and 1:20), and the analytical parameters compared with the standard curve were prepared in PBST to select the dilution factor in which the nonspecific interferences caused from the matrix were minimized.

#### Accuracy Studies

Blind spiked samples were prepared in the TSB culture broth and measured using the above-described ELISA. The samples were measured in triplicates, and the experiment was repeated on three different days.

## Supporting Information Available

General chemical and immunochemical methods and instruments; buffers; polyclonal antibody development; peptide synthesis [AIP-I–IV, AIPIVNH_(SH)_, and AIPIVS(NHS)] and characterization; theoretical calculations about immunogen’s structure; ELISA development and characterization; and data related to the growth of the distinct *S. aureus* strains analyzed (PDF)
