# Supplementary material for: Direct Quantitative Immunochemical Analysis of Autoinducer Peptide IV for Diagnosing and Stratifying Staphylococcus aureus Infections
Source: ACS Infect Dis. 2022 Feb 17;8(3):645–56. doi: 10.1021/acsinfecdis.1c00670 (PMC8922274; doi:10.1021/acsinfecdis.1c00670)
Supplement: Supplementary file 1 — id1c00670_si_001.pdf [file id1c00670_si_001.pdf]

SUPPORTING INFORMATION

Direct Quantitative Immunochemical Analysis of the Autoinducer Peptide IV (AIP-IV) for Diagnosing and Stratifying *Staphylococcus aureus* infections

Enrique-J. Montagut[A,B], Gerardo Acosta[C,B], Fernando Albericio[B,C,G,H], Miriam Royo[B,C], Gerard Godoy-Tena[D], Alicia Lacoma[D,E], Cristina Prat[D,E,F], , Juan-Pablo Salvador[A,B] and María-Pilar Marco[A,B]*

A. Nanobiotechnology for diagnostics (Nb4D), Department of Surfactants and Nanobiotechnology, Institute for Advanced Chemistry of Catalonia (IQAC) of the Spanish Council for Scientific Research (CSIC), 08750-Barcelona, Spain.

B. CIBER de Bioingeniería, Biomateriales y Nanomedicina (CIBER-BBN), 28029-Madrid, Spain.

C. Multivalent Systems for Nanomedicine (MS4N), Department of Surfactants and Nanobiotechnology, Institute for Advanced Chemistry of Catalonia (IQAC) of the Spanish Council for Scientific Research (CSIC), 08750-Barcelona, Spain.

D. Servei de Microbiologia, Hospital Universitari Germans Trias i Pujol, Institut d'Investigació Germans Trias i Pujol, Universitat Autònoma de Barcelona, 08916-Badalona, Spain.

E. CIBER de Enfermedades Respiratorias (CIBERES), 28029-Madrid, Spain.

F. Julius Center for Health Sciences and Primary Care, University Medical Center Utrecht, Utrecht University, 3584-Utrecht, The Netherlands.

G. Department of Organic Chemistry, Faculty of Chemistry, University of Barcelona, 08028-Barcelona, Spain

H. School of Chemistry and Physics, University of KwaZulu-Natal, 4000- Durban, South Africa.

It contains 16 pages, 11 Figures and 5 Tables

*To whom correspondence should be sent:

Prof. María- Pilar Marco
Nanobiotechnology for Diagnostics (Nb4D) group
Department of Surfactants and Nanobiotechnology
IQAC-CSIC
Jordi Girona 18-26, 08034 Barcelona, Spain
Spain
Phone: + 34 93 4006184
FAX: + 34 93 2045904
*E-mail: pilar.marco@cid.csic.es

GENERAL METHODS AND INSTRUMENTS:

Chemistry. Reagents and solvents were purchased from commercial sources and were used without further purification. HPLC-PDA-MS analyses were performed on a Waters Alliance 2795 with an automated injector and a photodiode array detector Waters 2996 coupled to an electrospray ion source (ESI-MS) Micromass ZQ mass detector, and the MassLynx 4.1 software. The instrument was operated in the positive ESI (+) ion mode. All the HPLC-PDA-MS analyses were carried out using the elution conditions: XSelect^{TM} C_{18} column (4.6 mm×50 mm, 3.5 μm). Elution solvent system: A: 0.1% HCOOH in H_2O and B: 0.07% HCOOH in CH_3CN. Gradient: 5 %B to 100 % B over 4.5 min at a flow rate of 2.0 mL/min. λ= 220 nm. HPLC-PDA analyses were performed on a Waters Alliance 2695 with a photodiode array detector Waters 2998 using elution conditions: XBridge^{TM} BEH130 C_{18} (3.5 μm, 4.5mm x 100mm) column. Elution solvent system: A: 0.045% TFA in H_2O and B: 0.036% TFA in CH_3CN. Flow rate of 2.0 mL/min and temperature 25°C. λ=220nm. Gradient A: 25 %B-65 %B in 8 minutes. Gradient B: 5 %B-100 %B in 8 minutes. Gradient C: 20 %B-100 %B in 8 minutes. UPLC analyses were performed on an Acquity high class (PDA detector, sample manager FNT and Quaternary solvent manager), using elution conditions: Acquity BEH C18 (1.7 μm, 2.1 mm X 50 mm) column. Elution solvent system: A: 0.045% TFA in H2O and B: 0.036% TFA in CH3CN. Flow rate of 0.61 mL/min and temperature 40° C. λ= 220 nm. Gradient A: 20 %B-65 %B in 2 minutes. Gradient B: 20 %B-40 %B in 2 minutes. Purification of the peptides was carried out on Waters semi-preparative HPLC-UV-MS, a separation module 2545, 2487UV detector with dual absorbance coupled to an electrospray ion source (ESI-MS) Micromass ZQ mass detector and a 2767 injector-manifold., using elution conditions: XBridge BEH 130 Semi-preparative C18 (5 μm, 19x100 mm) column. Elution solvent system: A: 0.1% HCOOH in H2O and B and B: 0.07% HCOOH in CH3CN at a flow rate of 16 mL / min. λ=220 nm. Gradient was specific for each peptide. HR-MS analysis were performed on an instrument LC/MSD-TOF Agilent Technologies G169A. Eluent: H2O:CH3CN (1:1) 200 [illegible]L/min. EM ESI-TOF(+)Frag. 175V.

Immunochemistry. Chemicals and biochemicals were obtained from Aldrich Chemical Co. (Milwaukee, WI, USA) and from Sigma Chemical Co. (St. Louis, MO, USA). The stock solutions of the AIPs (I to IV) used as standards were prepared in DMSO at 10 mM and stored at -20°C, then transferred to 4°C prior to their use. Purification of conjugates was carried out in ÄKTA Prime Plus using 2 HiTrap desalting columns both from GE Healthcare (Chicago, IL, USA) or either by dialysis using Spectra/Por membranes from Spectrumlabs (Piraeus, Greece, EU) with molecular

weight cut-off of 12-14 kDa. The matrix-assisted laser desorption ionization time-of-flight mass spectrometer (MALDI-TOF-MS) was a Bruker autoflex III Smartbeam spectrometer (Billerica, Massachusetts). Hapten densities of the conjugates were calculated by MALDI-TOF-MS by comparing the molecular weight recorded on the MALDI spectra of the native proteins to that of the BSA-SIA and HHQ-BSA bioconjugates. For this purpose, the bioconjugates were mixed with the freshly prepared matrix ((trans-3,5-dimethoxy-4-hydroxycinnamic acid, 10 mg mL^{-1} in 70:30 CH_3CN/H2O, 0.1% HCOOH) following the ''sandwich'' sample preparation method. The bioconjugate aliquot is diluted ½ using CH_3CN with HCOOH 0.2%. According to it, the matrix (2 µL) is deposited on the MALDI plate and dried, followed by the bioconjugate solution (2 µL, 2 to 5 mg mL^{-1} in 1:1 CH_3CN/H2O, 0.1% HCOOH), allowed to dry again and finally, the matrix solution (2 µL) was added over again. The resulting dried spot was then analyzed by MALDI-TOF-MS. Hapten densities were calculated through the equation: [MW(conjugate) − MW(native protein)]/[MW(hapten)-MW(lost atoms)]. The pH and the conductivity of all buffers and solutions were measured with a pH-meter pH 540 GLP and a conductimeter LF 340, respectively (WTW, Weilheim, Germany). Polystyrene microtiter plates used for the ELISAs were purchased from Nunc (Maxisorp, Roskilde, Denmark). Dilution plates were purchased from Nirco (Barberà del Vallés, Spain). Washing steps were performed on a Biotek ELx465 (Biotek Inc.). Absorbances were read on a Thermo Scientific MultiSkan GO (Thermo Fisher Scientific, Waltham, MA, USA) at a single wavelength mode (450 nm). The competitive curves were analyzed with a four-parameter logistic equation using the software GraphPad Prism 7.0 (GraphPad Software Inc., San Diego, CA, USA) according to the following formula: $y = B(A-B)/[1 - (x/C)^D]$, where A is the maximum absorbance, B is the minimum absorbance, C is the concentration producing 50% of the maximal absorbance, and D is the slope at the inflection point of the sigmoid curve. Unless otherwise indicated, the data presented correspond to the average of at least two well replicates.

Buffers. Unless otherwise indicated, phosphate buffer saline (PBS) corresponds to 10mM phosphate buffer and 0.8% saline solution (pH 7,5). Coating buffer is a 50 mM bicarbonate-carbonate buffer (pH 9,6). PBST is PBS with 0,05% Tween 20 (pH 7,5). Citrate buffer corresponds to a 40 mM sodium citrate solution (pH 5,5). The substrate solution contains 0,01% of 3,3',5,5'-tetramethylbenzidine (TMB) and 0,004% H_2O_2 prepared in citrate buffer. Borate buffer is 0,2 M sodium borate/boric acid (pH 8,7). All buffers were prepared using ultra-pure Milli-Q® water with a resistivity between 16-18 MΩ cm.

Polyclonal antisera (PAb). Six female New Zealand white rabbits weighing 1–2 kg were immunized with AIPIVNH-HCH (x3) or AIPIVS-HCH (x3) following the established protocols in the research group. Immunizations were carried out in the animal facility of the Research and Development Center (CID) of the Spanish Research Council (CSIC) Registration Number: B9900083, employing approved procedures that avoid unnecessary treatments and minimize suffering of the animals. The protocol used in accordance with the institutional guidelines under a license from the local government (DAAM 7463) and approved by the Institutional Animal Care and Use Committee at the CID-CSIC. The antisera (As) obtained by immunization with AIPIVNH-HCH were named As376, As377 and As378 and the As obtained by immunization with AIPIVS-HCH were named As379, As380 and As381. The animals were exsanguinated after 6 immunizations, and the final blood was collected in vacutainer tubes provided with a serum separation gel. Antisera were obtained by centrifugation at 4 °C for 10 min at 10 000 rpm, then stored at −80 °C in the presence of preservative 0.02% sodium azide. The antibody titer was assessed during the immunization process through non-competitive indirect ELISA. Microtiter plates were coated with a fixed concentration of AIPIVNH-BSA or AIPIVS-BSA conjugates (1 mg mL^{-1}) and the avidity of the produced antibodies was measured by preparing serial dilutions of the corresponding As.

PEPTIDE SYNTHESIS:

Synthesis of the AIPs-(I-IV), AIP-IVS(X) and AIP-IVNH linear precursors. Linear peptides precursors were synthesized manually on solid phase using 0.5 g of 2-chlorotrityl chloride (CTC) resin (1.6 mmol Cl/ g). The resin was conditioned with washings with dichloromethane (DCM) and dimethylformamide (DMF). The C-terminal residue was introduced by the addition of the corresponding Fmoc-aa-OH (1 mmol) and N,N-diisopropylethylamine (DIEA) (8.0 mmol) dissolved in anhydrous DCM on to the solid support. After 1.5 h MeOH (0.8 mL/g de resina, 400 µL) was added to block the unreacted chloride groups and the mixture was left to react 30 min. Then, it was filtered and the resin washed with DMF and DCM. Peptides were elongated by sequential Fmoc removal and coupling cycles using a Fmoc/tBu protecting group strategy. All functional groups of the amino acid side chains were protected with tert-butyl (tBu) group with the exception of the Cys thiol, which was protected with a 4-methoxytrityl group (Mmt). Fmoc removal was carried out by treatments (2 x 5 min) with piperidine-DMF solution (2:8, v/v). Amino acid couplings were performed by the addition of Fmoc-aa-OH (3 equiv.), *N*,*N*'-diisopropylcarbodiimide (DIC) (3 equiv.) and ethyl cyano(hydroxyimino)acetate (OxymaPure) (3 equiv., in DMF during 45 min. After each Fmoc removal or coupling steps the resin washed

with DMF, DCM and DMF. The last amino acid introduced was protected at the α-amino with the Boc protecting group.

For the linear precursor of the AIPIVNH erivative, Fmoc-Dap(Alloc)-OH was used instead of Fmoc-Cys(Mmt)-OH, and linear precursor was elongated with 8-(9-fluorenylmethyloxycarbonyl-amino)-3,6-dioxaoctanoic acid and Boc-Cys-(Trt)-OH. Once the peptide elongation was finished, the Alloc group was eliminated by treatments (3 x 15 min) with tetrakis(triphenylphosphine)-palladium(0) (0.1 equiv., 26 mg, ($Pd(Ph_3P)_4$) and phenylsilane (5 equiv., 140 μL, $PheSiH_3$) in anhydrous DCM.

The AIP-I to IV and lactam AIP-IV linear precursor peptidyl-resins were cleaved using mild acidolysis with TFA-H_2ODCM (2:4:94, v/v; six treatments of 1 minute/each). Then, the cleavage mixture was filtered and collected over H_2O and DCM was eliminated under vacuum. The final protected linear AIP-I to IV peptide solutions were lyophilized and used for cyclization without previous purification.

AIP IV cyclization. 104.4 mg (77.3 μmol) of AIP IV protected linear peptides were dissolved in 78 mL of DMF and PyBOP (52 mg, 1.3 equiv., 100 μmols) and DIEA (17 μL, 1.3 equiv., 100 μmol) were added. Thiolactone formation was controlled by HPLC-UV-MS and once the starting material was consumed, the DMF was eliminated under vacuum. The residue was dissolved in H_2O-CH_3CN (1: 1, v/v) and lyophilized, yielding 100 mg of protected AIP-IV crude. Protecting groups were eliminated by acidolysis with TFA-H_2O-TIS (95:2.5:2.5, v/v/v) at room temperature during 1h. Then, peptide was precipitated by adding slowly this solution to cold diethyl ether (Et_2O). The solid was washed 3 times with cold Et_2O, dissolved in H_2O-CH_3CN and lyophilized. 65 mg of crude AIPIV were obtained. Peptide was purified by semi-preparative HPLC-UV-MS getting 14.3 mg of pure AIP-IV. HPLC-PDA (gradient A): t_R: 5.977 min. Chemical purity: 94.94% (estimated by UV at 220nm). HR-MS: Calculated mass for $C_{48}H_{64}N_8 0_{12}S_2$: 1008.4085; found: $[M+H]^+$= 1009.4150; $[M+Na]^+$ = 1031.3978; $[M+K]^+$ = 1047.3716.

AIPs I-III cyclization. The cyclization of AIP I-III followed the process described below for AIP IV.

AIP-I: 391 mg of AIP I crude. Purified by HPLC-UV-MS. AIP 1 pure: 25.9 mg. UPLC (gradient A): Gradient 20-65% of ACN t: 2min.t_R: 1.588 min. Chemical purity: 99.36% (estimated by UV at 220nm). HR-MS: Calculated mass for $C_{43}H_{60}N_8 0_{13}S_2$: 960.3721; found: 961.3781 $[M+H]^+$; 983.3595 $[M+Na]^+$; 999.3329$[M+K]^+$.

AIP-II: 395.2 mg of AIP II crude. Purified by HPLC-UV-MS. AIP 2 pure: 22.3 mg. UPLC (gradient B): t_R: 1.567 min. Chemical purity 96.07% (estimated by UV at 220nm). HR-MS: Calculated mass for $C_{38}H_{58}N_{8}0_{12}S_{2}$: 878.3956; found: 879.4022 $[M+H]^+$; 901.3853 $[M+Na]^+$; 917.3575$[M+K]^+$.

AIP-III: 122,5 mg of AIP 3 crude. Purified by HPLC-UV-MS. AIP 3 pure: 14.4 mg. UPLC (gradient A): t_R: 1.810 min. Chemical purity 90,56% (estimated by UV at 220nm). HR-MS Calculated mass for $C_{38}H_{58}N_{8}0_{10}S_{2}$: 818.3997; found: 819.4079 $[M+H]^+$; 841.3890 $[M+Na]^+$; 857.3636 $[M+K]^+$.

Synthesis of the AIPIVS(NHS) hapten. Equimolar quantities of AIP-IV and the NHS-PEG (2) -NHS) with DIEA (1 equiv.) in DMF were mixed. The evolution of the reaction was controlled by HPLC-MS, and once was finished DMF was eliminated under vacuum. The AIP-IV-PEG (2)–NHS was purified by semi-preparative HPLC getting 21.7 mg of final pure conjugate. HPLC-PDA (gradient C): t_R: 5.977 min. Chemical purity: 95.61% (estimated by UV at 220nm). HPLC-MS: calculated mass for $C_{60}H_{79}N_{9}O_{19}S_{2}$: 1293.49; found: $[M+H]^{+1}$ = 1294.5; $[M+NH_4]^{+1}$ = 1311.7; $[M+2H]^{+2}$ = 648.2.

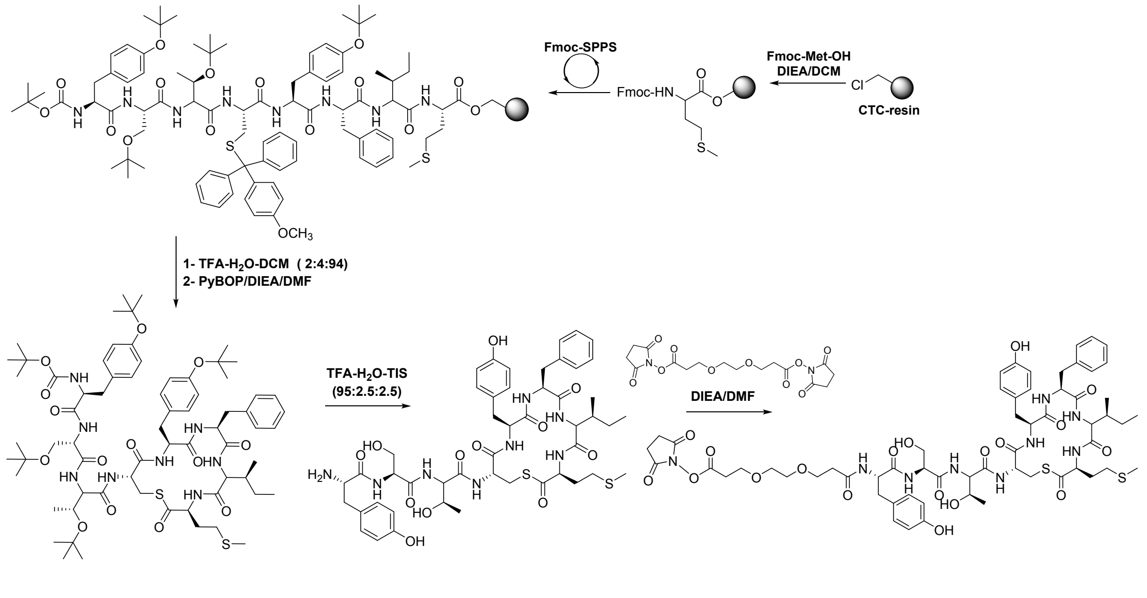

Figure S1. Schematic representation of the AIPIVS hapten synthesis.

AIPIVS(SH) and AIPIVS(NH2) haptens: Previous attempts to synthesize these haptens were addressed by reacting the N-terminal tyrosine of the linear AIPIV precursor with 8-amino-3,6-dioxooctanoic acid derivatized with a cysteine for the case of the AIPIVS(SH). However, in both cases after elimination of the Trt and Boc groups, the formation of cyclic peptides resulting from the attack of the terminal SH and NH2 groups of the spacer to the electrophilic carbonyl of the thiolactone was observed

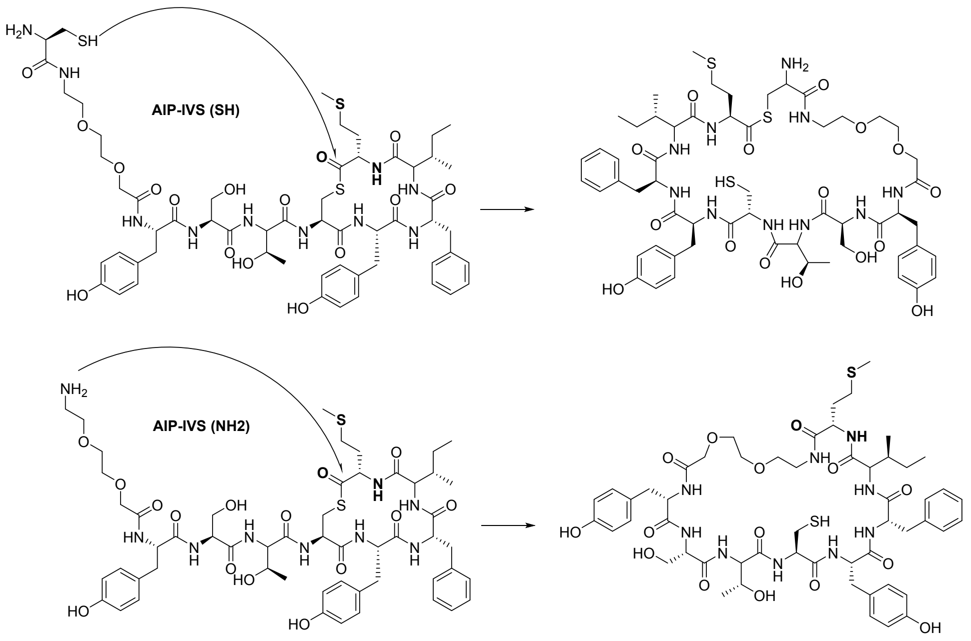

Figure S2. Spontaneous rearrangement of the proposed hapten AIPIVS(SH) and the synthetic intermediate of the AIPIVS(NH2) hapten after elimination of the PEG(2) amine protecting group.

Synthesis of the AIPIVNH(SH) hapten. The protected AIP-IV lactam linear peptide precursor (100 mg) was dissolved in DMF and PyBOP (34 mg, 65 μmols) and DIEA (15μL; 82 μmols) were added. The reaction was controlled by HPLC-MS. Once the reaction was finished, DMF was eliminated under vacuum. Then, protecting groups were eliminated by acidolysis with TFA:H_2O:TIS (95:2.5:2.5, v/v/v) at room temperature during 1h. Then, peptide was precipitated by adding this solution slowly to cold Et_2O. The solid was washed 3 times with cold Et_2O, dissolved in H_2O:CH_3CN and lyophilized. 65mg of AIP-IV lactam derivative (AIPIVNH) were obtained. After purification by semi-preparative HPLC-UV-MS, a mixture 24.5 mg monomer and dimer (3/1) was obtained. The dimer was reduced with a treatment with tris(2-carboxyethyl)phosphine (TCEP) and used for conjugation without further purification. HPLC-PDA (gradient B): t_R: 5.572 min. Chemical purity: 98.39% (estimated by UV at 220nm). HR-MS: Calculated mass for $C_{57}H_{81}N_{11}0_{16}S_2$: 1239.5304; found: 1240.5397 $[M+H]^1$; 1262.5209 $[M+Na]^+$; 1278.4911 $[M+K]^{+1}$.

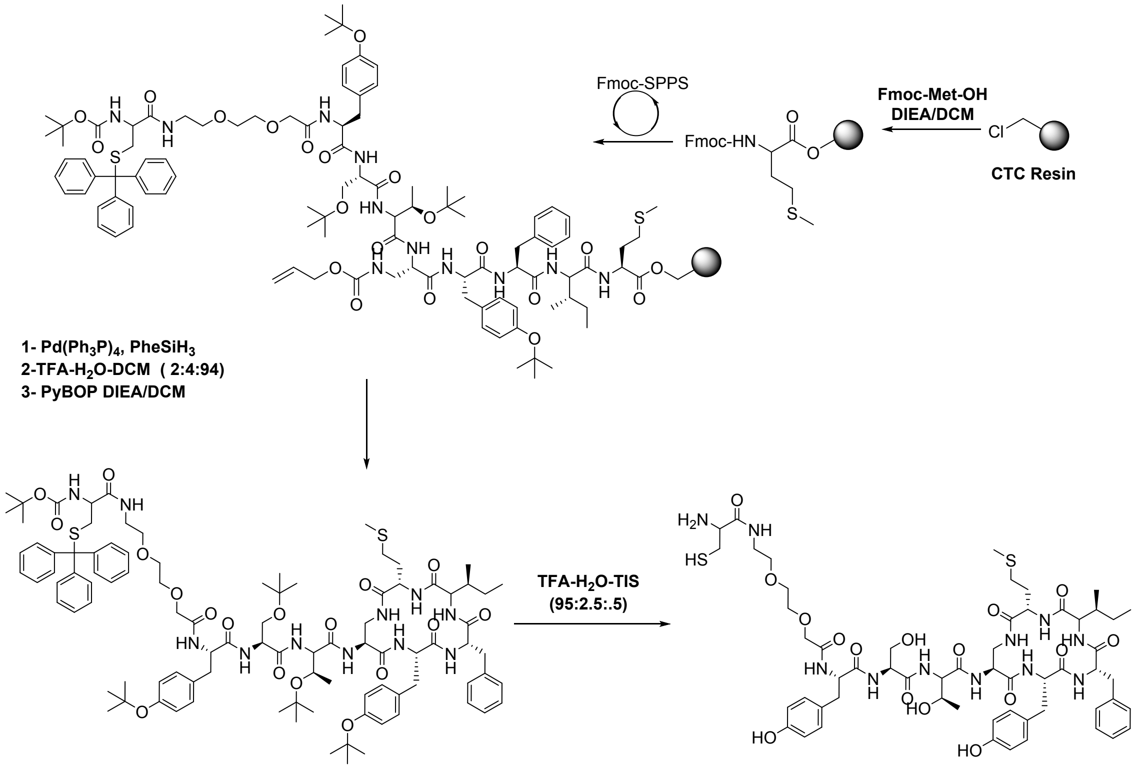

Figure S3. Schematic representation of the AIPIVNH hapten synthesis.

CHROMATOGRAPHIC AND SPECTROMETRIC DATA

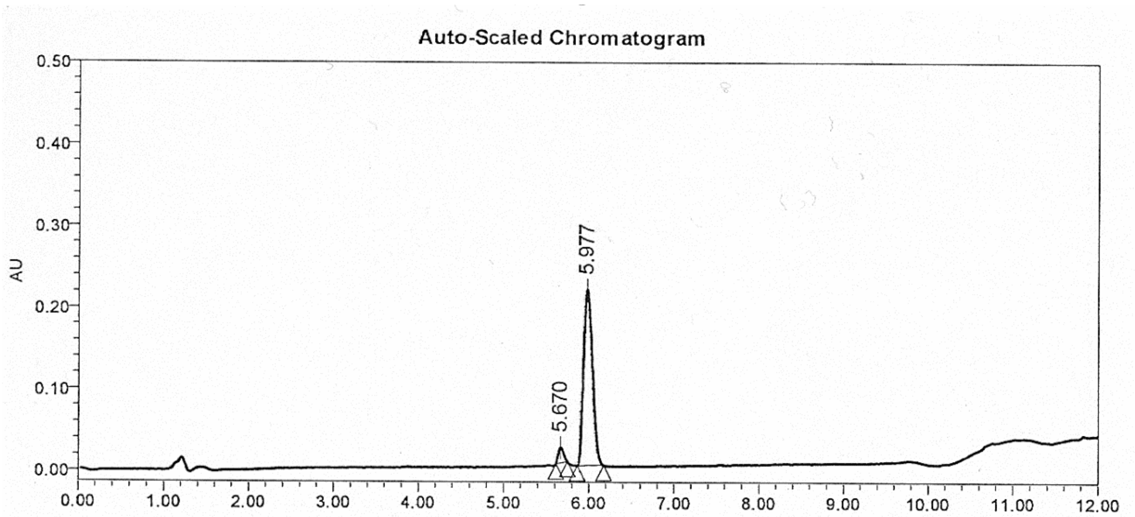

Figure S4. HPLC-PDA chromatogram of synthetic AIP-IV

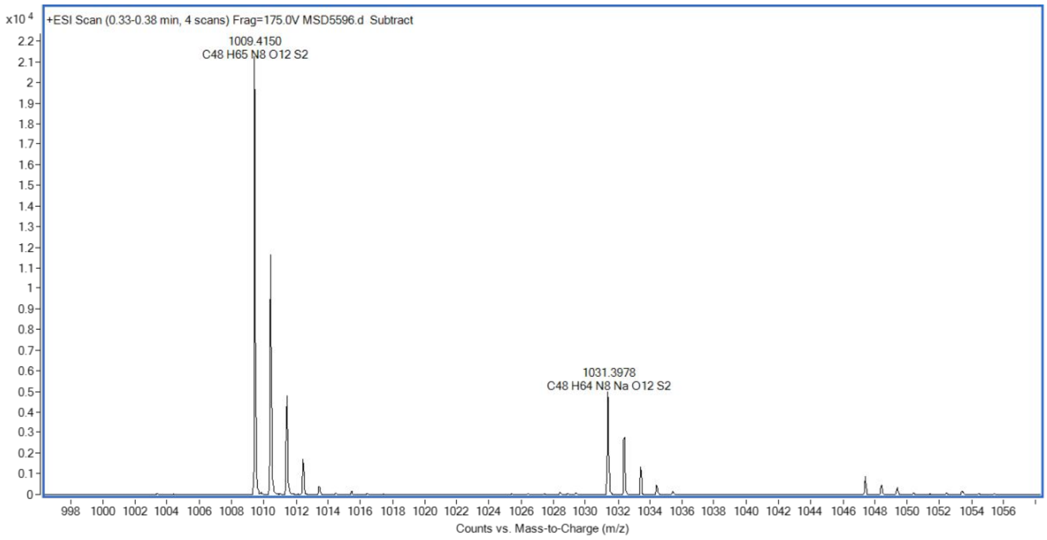

Figure S5. HR-MS Spectra of Synthetic AIP-IV.

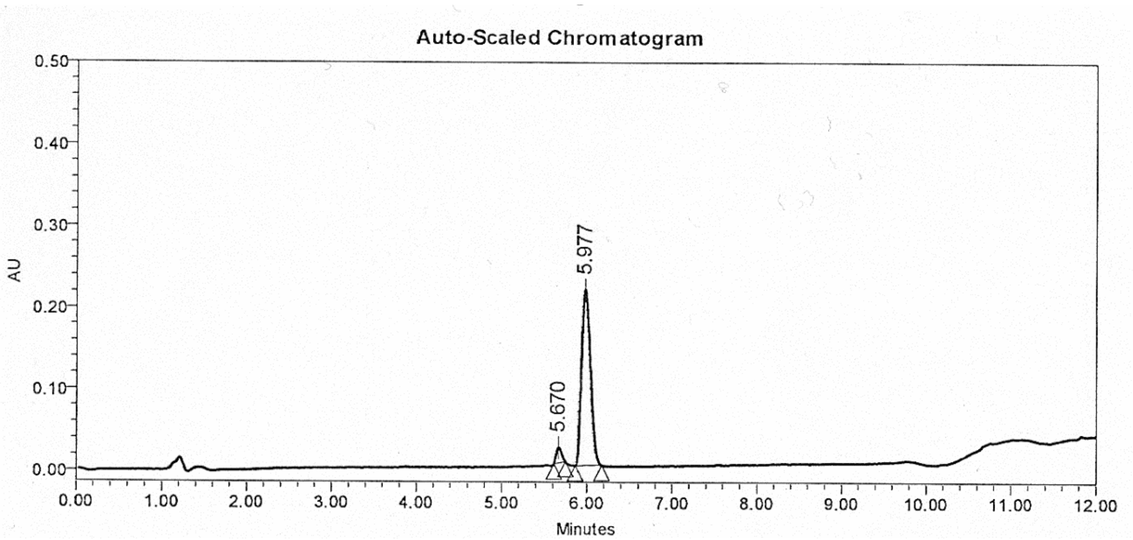

Figure S6. HPLC-PDA chromatogram of AIPIVS(NHS) hapten.

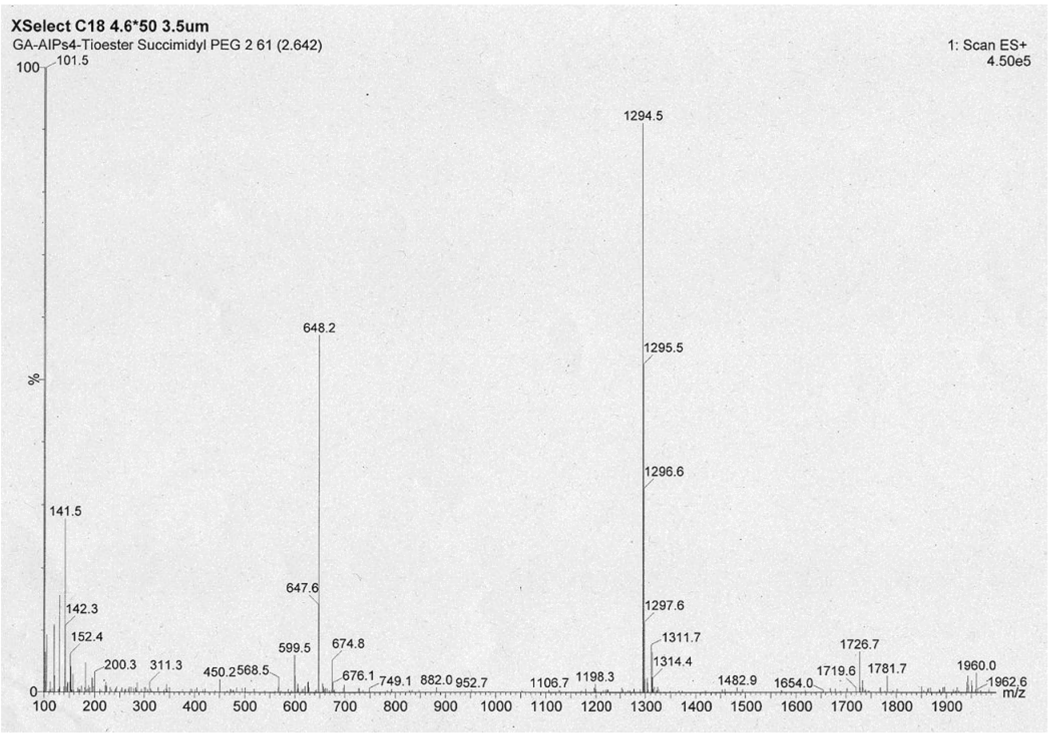

Figure S7. MS (ESI) spectra from HPLC-PDA from AIPIVS(NHS) hapten.

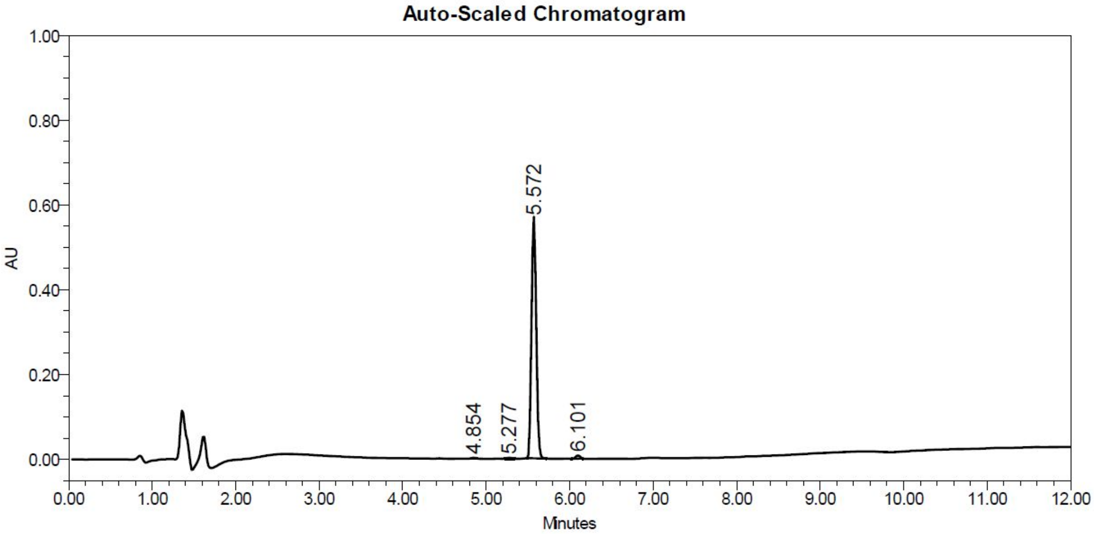

Figure S8. HPLC-PDA chromatogram of AIPIVNH(SH) hapten.

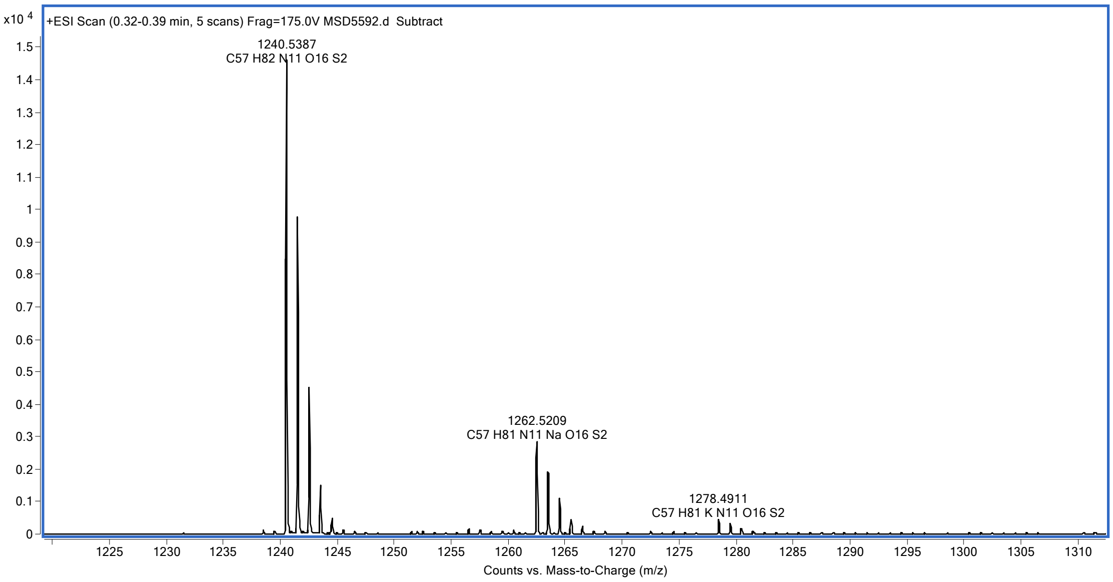

Figure S9. HR-MS spectra of AIPIVNH(SH) hapten

CHARACTERIZATION OF THE BIOCONJUGATES

Table S1. Data on the bioconjugation yield and hapten densities of the AIP-IV bioconjugates.

	Quantity (mg)	***Yield (%)***	***Hapten density***
**AIPIVS-BSA**	**3.80**	**76.0**	**8**
**AIPIVS-HCH**	**4.49**	**89.8**	**-**
**AIPIVNH-BSA**	**3.33**	**67.0**	**6**
**AIPIVNH-HCH**	**3.29**	**65.8**	**-**

Hapten densities of BSA conjugates were calculated from MALDI-TOF-MS/MS analysis. HCH conjugate could not be analyzed because of the large molecular weight. For this purpose, BSA ans HCH bioconjugates were prepared in paralell, under exactly the same conditions. The data obtained with the corresponding BSA bioconjugates was used as bioconjugation control.

DEVELOPMENT OF THE ELISA:

Non-competitive indirect two-dimensional titration experiments. Non-competitive indirect ELISA were carried out to select the concentrations of coating antigen (CA) and the As dilutions more suitable for the competitive assays. For this purpose, the binding serial dilutions of the antisera (1/1000 to 1/64000, and zero in PBST, 100 µL/well) to microplates coated with the BSA

bioconjugates (5 µg mL^{-1} to 5 ng mL^{-1}, and zero in coating buffer, 100 µL/well) was measured. From these experiments, optimum concentrations for coating antigens and antisera dilutions were chosen to produce around 1.0-1.5 units of absorbance after 30 min of competitive step.

Table S2. Analytical parameters of the best competitive indirect ELISAs for AIP-IV.

**Immunogen➢**	**AIPIVNH(SH)-HCH**			**AIPIVS(NHS)-HCH**	
	**As376; 1/64000**	**As377; 1/12000**	**As378; 1/16000**	**As379; 1/16000**	**As380; 1/4000**
**[CA]*^b^* (µg/ml)**	0.17	0.63	0.63	0.45	1.25
Bottom	0.04	0.07	0.12	0.02	0.23
Top	0.83	1.56	1.35	1.57	1.54
Hill Slope	-0.80	-0.81	-090	-0.78	-0.93
IC_{50}	186.7	3600	294.5	79.4	5.7
R^2	0.989	0.993	0.996	0.996	0.993

[a]The table shows only the best competitive assays obtained after the firts screening of the diferent antibodies raised against the AIPIVS(NHS) and AIPIVN(SH) immunizing haptens.

[b]The bioconjugates used as coating antígens are homologous to the immunogen (same hapten) as the analytical features were substantially better than those assays obtained under heterologous conditions. The As381 (AIPIVS(NHS)-KLH was not oble to provide a competitive assay for the detection of AIP-IV.

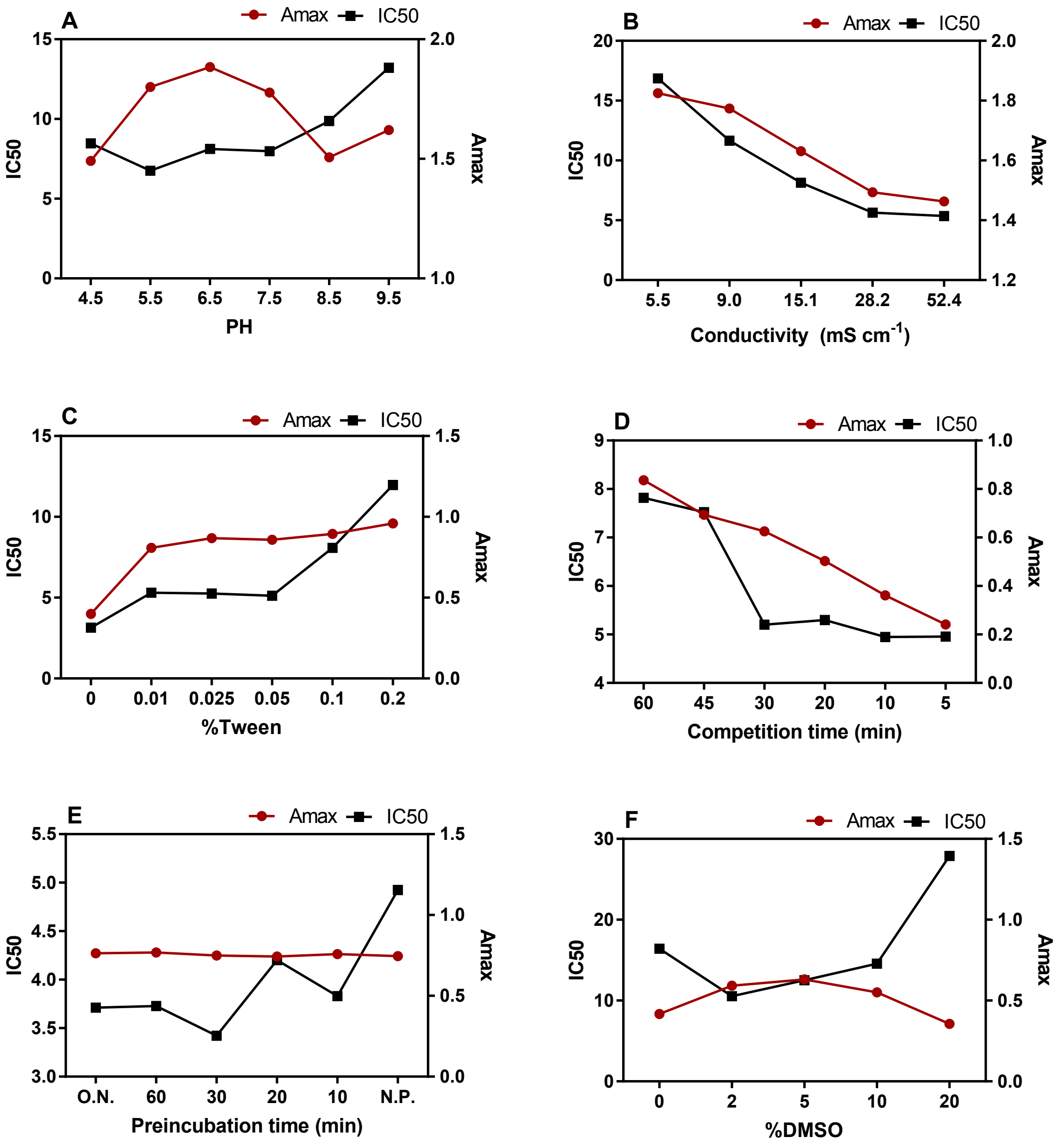

Figure S10. As380/AIPIVS-BSA ELISA performance in the physicochemical parameters optimization study. The selection of the most appropriate conditions (**Table S2.**) was based on the variations in Amax, IC50 and slope values (not shown) of the generated calibration curves providing better signal/noise ratio, detectability and sensitivity. The studied parameters were **A.** pH **B.** Ionic Strength **C.** % Tween 20 **D.** competition time **E.** preincubation time **F.** % organic solvent (DMSO). All the studies were performed by varying the composition of the buffer used in the competitive step or the antibody detection times. Eventually, the conditions providing better features were evaluated again separately and in conjunction.

Table S3. Physicochemical parameters selected for the As380/AIPIVS-BSA.

	**As380/AIPIVS-BSA**
**As dilution**	1/4000
**[Competitor] (µg mL^{-1})**	0.63
**pH**	7.5
**Conductivity (mS cm^{-1})**	15
**Tween 20 (%)**	0.05
**Competition time (min)**	30
**Preincubation time (min)**	0
**Organic solvent (%)**	0

The parameters improving the features of the assay were assessed separately and in conjunction.

Table 4S. Summary of the reported methods to analyze S. aureus AIPs and the detectability reached

**Peptide**	**Method**	**Detectability**	**Matrix**	**Ref**
**AIP-III**	UPLC-MS	Detected not quantified [a]	TSB	Roux et al, 2014[1]
**AIP-I**	UPLC-MS	LOD 0.0035 µM<br>LOQ 0.10 µM	TSB	Todd et al, 2016[2]
**AIPs**	UPLC-MS	LOD 0.063 uM	TSB	Gless et al., 2019[3]
**AIP-I**	UPLC-MS	LOD 0.25 µM	TSB	Junio et al., 2013[4]

Table S5. Optical density and Colony Forming Units count for the extracted aliquots of TSB culture broth samples at the selected times of growth for the clinical isolates and reference strains used in this work.

		3_40448		6_19850		32_75664		48_86474		165_36759		197_63535		Newman		USA300	
#	Time	OD_{600}	CFU/ml	OD_{600}	CFU/ml	OD_{600}	CFU/ml	OD_{600}	CFU/ml	OD_{600}	CFU/ml	OD_{600}	CFU/ml	OD_{600}	CFU/ml	OD_{600}	CFU/ml
0	0	0,09	0,16	0,09	0,33	0,09	0,06	0,12	0,46	0,08	0,36	0,09	0,30	0,08	0,10	0,07	0,23
1	1	0,28	0,46	0,31	0,90	0,29	0,55	0,27	0,53	0,214	0,73	0,21	1,02	0,25	0,50	0,20	0,86
2	2	0,96	2,06	0,87	1,40	0,84	1,86	0,67	1,40	0,736	2,63	0,84	1,70	0,73	1,80	0,71	1,76
3	3	1,50	3,58	1,37	4,00	1,43	3,97	1,28	5,50	1,32	6,80	1,48	4,90	1,38	5,60	1,36	2,20
4	4	1,78	7,00	1,74	8,00	1,70	8,50	1,60	8,00	1,63	12,00	1,72	8,30	1,68	5,60	1,62	8,60
5	5	1,87	7,03	1,84	17,17	1,87	11,50	1,73	12,10	1,77	14,70	1,79	15,00	1,75	12,30	1,72	8,70
6	6	1,89	13,60	1,89	19,00	1,89	7,60	1,79	6,70	1,86	4,30	1,78	7,30	1,82	9,00	1,71	9,60
7	9	1,88	11,50	1,92	12,66	1,95	13,02	1,84	13,80	1,93	5,50	1,82	9,30	1,73	8,60	1,75	10,00
8	12	1,84	8,90	1,86	9,97	1,89	11,01	1,81	13,50	1,96	17,00	1,84	17,10	1,73	7,70	1,63	9,30
9	15	1,82	11,30	1,73	11,60	1,89	15,00	2,05	15,00	2,06	20,6	1,97	13,3	1,78	6,5	1,72	7,6
10	18	1,89	8,00	1,85	12,30	1,95	8,00	1,91	8,00	2,02	15,00	1,93	13,40	1,70	6,70	1,73	8,00
11	21	1,76	9,00	1,83	9,00	1,89	17,30	1,96	117,30	2,02	24,00	1,94	18,00	1,90	12,60	1,81	11,60
12	24	1,97	15,60	1,91	22,00	1,81	9,00	2,04	9,00	2,05	25,30	1,96	19,00	2,01	13,00	1,77	14,60
13	48	1,91	-	-	-	2,04	-	1,88	-	1,96	-	1,87	-	1,89	-	1,82	-

OD_{600} is expressed in nm; CFUs are 1 x 10^8

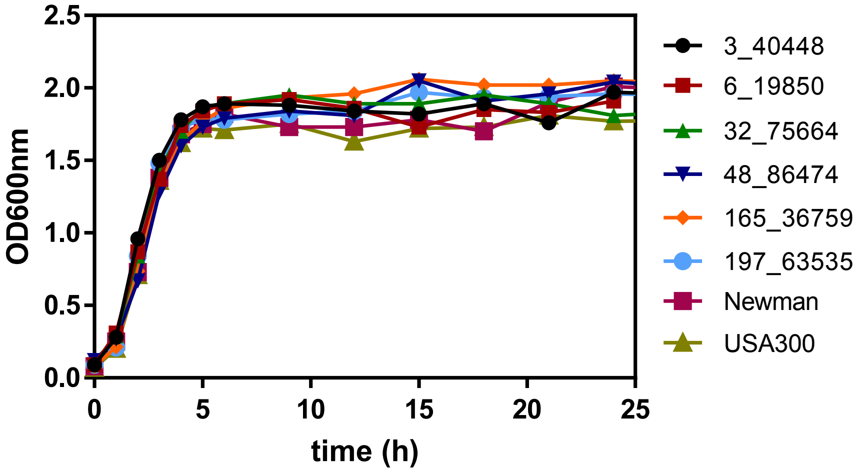

Figure S11. Growth curves of the different clinical isolates and reference strains recorded based on monitoring the absorbance at 600 nm (OD600).

Literature cited

1. Roux, A.; Todd, D. A.; Velázquez, J. V.; Cech, N. B.; Sonenshein, A. L., CodY-Mediated Regulation of the Staphylococcus aureus Agr System Integrates Nutritional and Population Density Signals. *Journal of Bacteriology* **2014,** *196* (6), 1184-1196.
2. Todd, D. A.; Zich, D. B.; Ettefagh, K. A.; Kavanaugh, J. S.; Horswill, A. R.; Cech, N. B., Hybrid Quadrupole-Orbitrap mass spectrometry for quantitative measurement of quorum sensing inhibition. *Journal of Microbiological Methods* **2016,** *127*, 89-94.
3. Gless, B. H.; Bojer, M. S.; Peng, P.; Baldry, M.; Ingmer, H.; Olsen, C. A., Identification of autoinducing thiodepsipeptides from staphylococci enabled by native chemical ligation. *Nature Chemistry* **2019,** *11* (5), 463-469.
4. Junio, H. A.; Todd, D. A.; Ettefagh, K. A.; Ehrmann, B. M.; Kavanaugh, J. S.; Horswill, A. R.; Cech, N. B., Quantitative analysis of autoinducing peptide I (AIP-I) from Staphylococcus aureus cultures using ultrahigh performance liquid chromatography–high resolving power mass spectrometry. *Journal of Chromatography B* **2013,** *930*, 7-12.
5. Lubkowicz, D.; Ho, C. L.; Hwang, I. Y.; Yew, W. S.; Lee, Y. S.; Chang, M. W., Reprogramming Probiotic Lactobacillus reuteri as a Biosensor for Staphylococcus aureus Derived AIP-I Detection. *ACS Synthetic Biology* **2018,** *7* (5), 1229-1237.
